# Spatially Resolved Quantification of Chromatin Condensation through Differential Local Rheology in Cell Nuclei Fluorescence Lifetime Imaging

**DOI:** 10.1371/journal.pone.0146244

**Published:** 2016-01-14

**Authors:** Stephen T. Spagnol, Kris Noel Dahl

**Affiliations:** 1 Department of Chemical Engineering, Carnegie Mellon University, 5000 Forbes Ave., Pittsburgh, Pennsylvania, 15213, United States of America; 2 Department of Biomedical Engineering, Carnegie Mellon University, 5000 Forbes Ave., Pittsburgh, Pennsylvania, 15213, United States of America; University of Michigan, UNITED STATES

## Abstract

The linear sequence of DNA encodes access to the complete set of proteins that carry out cellular functions. Yet, much of the functionality appropriate for each cell is nested within layers of dynamic regulation and organization, including a hierarchy of chromatin structural states and spatial arrangement within the nucleus. There remain limitations in our understanding of gene expression within the context of nuclear organization from an inability to characterize hierarchical chromatin organization *in situ*. Here we demonstrate the use of fluorescence lifetime imaging microscopy (FLIM) to quantify and spatially resolve chromatin condensation state using cell-permeable, DNA-binding dyes (Hoechst 33342 and PicoGreen). Through *in vitro* and *in situ* experiments we demonstrate the sensitivity of fluorescence lifetime to condensation state through the mechanical effects that accompany the structural changes and are reflected through altered viscosity. The establishment of FLIM for resolving and quantifying chromatin condensation state opens the door for single-measurement mechanical studies of the nucleus and for characterizing the role of genome structure and organization in nuclear processes that accompany physiological and pathological changes.

## Introduction

The structural state of DNA in the nucleus, corresponding to varying levels of chromatin condensation, is integral to its function. This varying of hierarchical condensation is thought to allow or prevent access of transcription factors to the linear sequence[1,2] while serving as a central feature of nuclear organization.[3] Chromatin states are broadly categorized into heterochromatin and euchromatin, owing to their historical association with the density of their appearance with light[4] or electron microscopy.[5] Heterochromatin is generally associated with highly condensed, gene-poor stretches of chromatin consistent with repression.[6] This dense packing of heterochromatin is driven in part by histone modifications particularly at lysine residues, including deacetylation and specific methylation patterns.[7] These modifications enhance the binding of histones and other chromatin architectural proteins that drive further condensation, such as heterochromatin protein 1 (HP1). Heterochromatin is further classified into constitutive heterochromatin that is very highly condensed and repressed, and facultative heterochromatin that is condensed but may become activated in response to environmental signals.[7] By contrast, euchromatin is gene-rich and largely decondensed, allowing for active processes including transcription.[8] Chromatin remodeling associated with decondensation is an active, ATP-dependent process that involves modification as well as movement or ejection of histone proteins.[9] The subtleties associated with these and other varying chromatin modification processes lead to gradations in condensation. Thus, the binary assignment of chromatin state is largely an oversimplification that obscures the reality of highly dynamic chromatin structure with rapid and frequent remodeling between intermediate states of condensation providing an element of plasticity to chromatin function.[7,10,11]

In addition to chromatin condensation state, there is a non-random three-dimensional arrangement of chromatin within the nucleus, which is thought to impact genome function and gene expression.[12,13] Proteins are heterogeneously distributed throughout the nucleus, giving rise to protein complexes that form distinct functional environments including Cajal bodies, PML bodies, nucleoli, transcription sites and many other subnuclear bodies wherein the spatial arrangement of chromatin becomes critical.[3] As such, the differential condensation state of chromatin throughout the nucleus is integral to these functional sites;[14] *e*.*g*., loops of decondensed chromatin are present in the interior of the nucleolus, and the nucleolus is enveloped by a border of heterochromatin.[15] By contrast, the spatially resolved condensation states of chromatin associated with other functional sites–including the appropriate length scales to be measured–remains to be determined. Of particular consequence is the inability to spatially resolve chromatin condensation state as it varies temporally with evolving processes, including the dynamic chromatin mobility that is intimately related to its condensation state.[16,17]

There are complementary ways to quantify and spatially resolve chromatin condensation state in human cell nuclei, but most have significant limitations. Resolution itself is typically restricted for intensity-based light microscopy methods.[18] Additionally, methods such as electron microscopy often require damaging fixation procedures. Fixation and disruption of structures can similarly reduce resolution, quantification and reproducibility for utilizing immunocytochemistry[19] and *in situ* hybridization techniques. Major advances in quantifying chromatin structure have been made using specialized cell lines with fluorescently labelled nucleosomal elements.[18] These methods have proven very useful, particularly when coupling fluorescence intensity measurements with other fluorescence-based measurements (including fluorescence anisotropy[20], fluorescence lifetime and/or Fӧrster resonance energy transfer[21]) that enhance spatial resolution. Similarly, spatial measurements of the diffusion of GFP-tagged histones and chromatin proteins as well as freely diffusing exogenous GFP monomers and oligomers have provided useful measures of chromatin condensation and reorganization.[22–24] However, overexpressed proteins can produce structural artifacts, and the use of specialized cell lines hinders its application to primary human cell lines where chromatin condensation and organization is tightly regulated most similarly to that observed *in vivo*.

Mechanical measurements that leverage the inherent relationship between mechanical-structural coupling of chromatin condensation states[25–27] as related to chromatin mobility experiments noted above have similarly been used to quantify chromatin condensation state *in situ*. Current mechanics-based methods, including particle tracking of fluorescent probes[16,28] or bulk mechanical measurements[25–27] overcome the limitation of specialized cell lines and can be used in live cells, but they are generally low-throughput and provide mostly ensemble information.

Here we utilize fluorescence lifetime imaging microscopy (FLIM) of a membrane permeable, DNA-binding fluorophore for quantifying and spatially resolving chromatin condensation state in primary human endothelial cells. The phenomena of fluorescence lifetime measures the exponential decay rate (via time) of a fluorophore from the its excited state to the radiative fluorescence emission.[29] The fluorescence lifetime is highly sensitive to the multiple aspects of local fluorophore environment within length scales of up to 10 nm and down to angstroms.[29,30] We show that differential chromatin condensation states within the nucleus uniquely contribute to the fluorescence lifetime. Further, through *in vitro* measurements of DNA solutions we demonstrate that fluorescence lifetime is acutely sensitive to local solution viscosity, and that it varies most significantly with DNA condensation state even in the absence of binding proteins. We highlight the utility of this technique for measuring large scale chromatin decondensation and reorganization in response to chemically-stimulated changes in gene expression through both quantification of these changes as well as demonstration of the spatially resolved distribution of chromatin condensation states in stimulated nuclei. We also show the high spatial resolution of FLIM through co-labeling distinct functional sites within the nucleus and resolving the characteristic chromatin condensation state around those regions. The establishment of FLIM for quantifying the spatial organization of chromatin condensation in primary human cell nuclei provides a potentially high-throughput technique for assaying the functionally-derived structural changes of chromatin through its unique dependence on mechanics. This will enable large-scale measurements of chromatin condensation state changes, including their temporal evolution, to better understand functional changes of nuclear processes.

## Results

### Differential Fluorescence Lifetime in Human Cell Nuclei

The fluorescence lifetime is mostly insensitive to properties of the incident light that lead to the initial excitation such as the exposure time, intensity and wavelength (including a range of single- or multi-photon wavelengths within the excitation spectrum) as well as the emission artifacts including fluorophore concentration and photobleaching.[29] Instead, the fluorescence lifetime depends on the local environment of the fluorophore.[29] We first aimed to investigate the fluorescence lifetime sensitivity to chromatin condensation state in human umbilical vein endothelial cell (HUVEC) nuclei for the minor groove DNA-binding fluorophore Hoechst 33342. Cells presented in the results were fixed and permeabilized, but fixation with formaldehyde has been shown to be the least restrictive means of fixation, leaving chromatin structure largely intact and allowing for DNA and nucleosome movement (see Methods).[31] We observed statistically similar fluorescence lifetime in live cell nuclei with Hoechst 33342, but analysis methods showed more variability (S1 File). This variability was due to cell translocation during the imaging as well as other temporal sampling effects associated active remodeling and evolution of chromatin condensation states with time.

**Fig 1:**
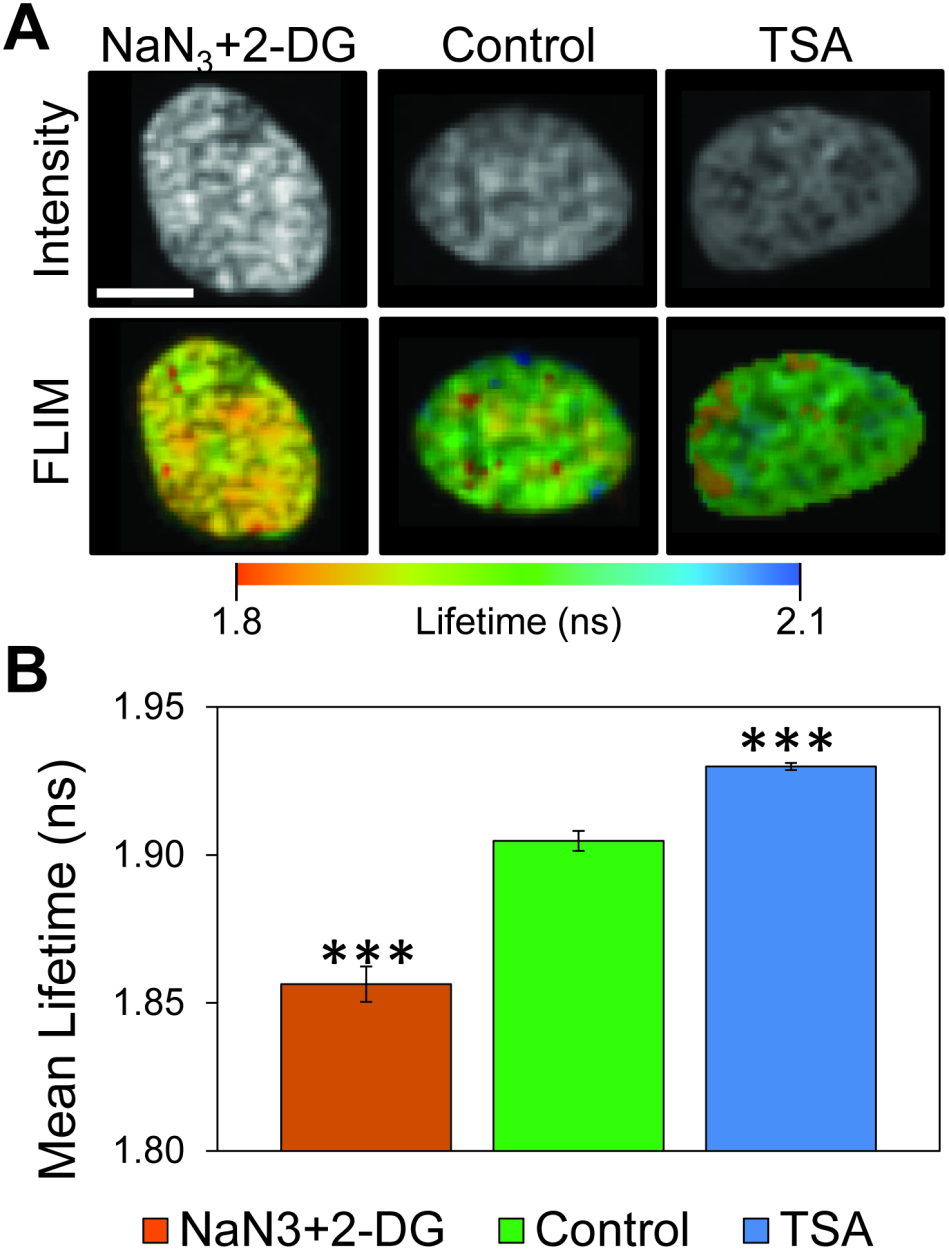
Fluorescence lifetime measurements of chromatin condensation state in human umbilical vein endothelial cell nuclei. (A) Fluorescence intensity confocal images (top) and mean fluorescence lifetime heat maps (bottom) of chromatin is measured in endothelial cell nuclei labeled with Hoechst 33342. Cells are treated with NaN_3_+2-DG for chromatin hypercondensation or with TSA for chromatin decondensation. Altered fluorescence intensity with treatments show differential chromatin condensation state, with more intense fluorescence arising from highly concentrated condensed chromatin. Mean fluorescence lifetime heat maps similarly indicate spatial arrangement of local fluorophore environments for labeled chromatin consistent with varying chromatin condensation state. Treatment with NaN_3_+2-DG results in more punctate regions of fluorescence intensity and shorter mean fluorescence lifetime (orange) relative to untreated controls, while TSA resulted in a significant reduction in punctate regions and longer mean fluorescence lifetime (blue). Scale bar is 10 μm. (B) The mean fluorescence lifetime of segmented nuclei for the various treatment conditions was calculated using Eq 2. Treatment with NaN_3_+2-DG resulted in a strong reduction in the mean fluorescence lifetime relative to untreated controls. By contrast, TSA treatment resulted in a dramatic increase in the mean fluorescence lifetime relative to untreated controls which indicated an increase in chromatin condensation state homogeneity throughout the cell nucleus. Error bars indicate standard error of the mean of pixel-to-pixel mean fluorescence lifetime differences of segmented nuclei in fields of view across multiple fields of view under each treatment condition (*** p<<0.001). Histograms and standard deviations are in S2 File.

Heat maps of the spatially resolved mean fluorescence lifetime indicate a broad distribution of fluorescence lifetimes throughout the control cell nucleus (Fig 1A, bottom middle panel) resulting from unique fluorophore environments. We decondensed chromatin with the histone deacetylase inhibitor Trichostatin A (TSA) and induced chromatin condensation by depleting ATP using sodium azide (NaN_3_) and 2-deoxyglucose (2-DG). Treatment with NaN_3_+2-DG lead to condensation of chromatin and punctate regions with a more homogeneous distribution of mean fluorescence lifetimes trending towards lower values (Fig 1A, bottom left panel). Decondensation of chromatin by treatment with TSA resulted in a similarly more homogeneous distribution of spatially resolved mean fluorescence lifetime in the heat maps, but instead trending towards higher mean fluorescence lifetimes (Fig 1A, bottom right panel). By contrast, the control cell nucleus exhibited a wide distribution of high and low values ranging between both extremes (Fig 1A, bottom middle panel and Fig A in S1 File). The heterogeneity and variance of the samples, including the very low lifetimes associated with hyperaggregation (0.6 and 1.3 ns) are visible in the histograms of fluorescence lifetime (S2 File).

For treatment to treatment comparison, we quantified these changes by calculating the mean fluorescence lifetime using Eq 2 for all segmented nuclei throughout each field of view, and the standard errors include the nucleus-to-nucleus variation as well as the pixel to pixel variability from regions of differential compaction (see Methods for statistical comparison). Chromatin decondensation resulted in an increase in the mean fluorescence lifetime, while chromatin condensation resulted in a significant reduction (Fig 1B).

Additionally, TSA treatment resulted in a statistically significant reduction in variance (S2 File), indicating more uniform chromatin condensation state throughout the nucleus as measured by the mean fluorescence lifetime. We repeated fluorescence lifetime experiments using an intercalating DNA dye, PicoGreen and found similar changes in lifetime between nuclei with TSA decondensed chromatin and control samples (S3 File). Thus, the fluorescence lifetime of chromatin-bound DNA probes (including Hoechst 33342) indicate local chromatin condensation state in human cell nuclei.

### Fluorescence Lifetime Sensitivity of λ-DNA

Having established a relationship between chromatin condensation and fluorescence lifetime, we aimed to determine the environmental effectors driving the change in the fluorescence lifetime of the fluorophore bound to DNA. The fluorescence lifetime is known to depend on factors such as viscosity, polarity, temperature and the presence of quenchers in the surrounding medium local to the fluorophore.[29] We examined the fluorescence lifetime dependence on in vitro λ-DNA associated with (i) compaction changes induced by molecular crowding (ii) polarity changes from divalent charge and (iii) viscosity changes from high molecular weight solvents.

**Fig 2:**
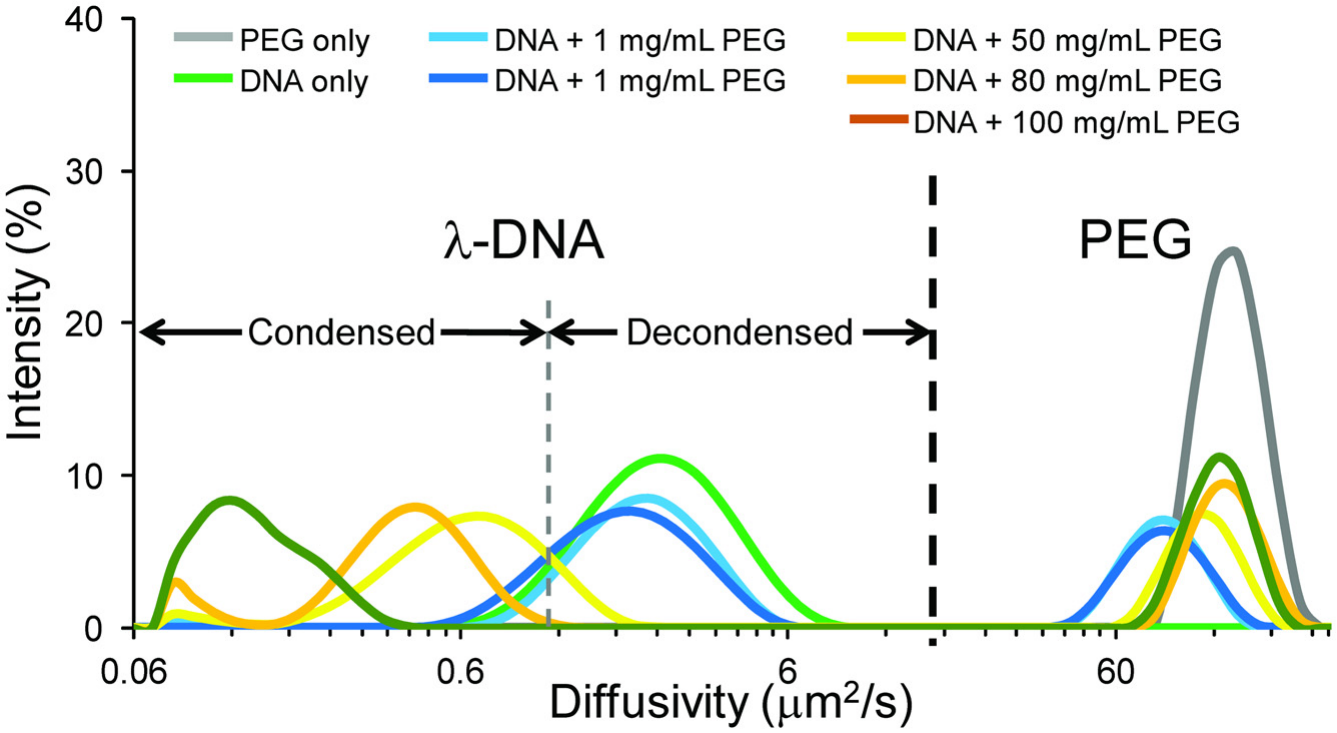
Dynamic light scattering measurements of *in vitro* λ-DNA solutions of varying condensation state. Measurements of PEG 6000 (gray) and λ-DNA (green) alone indicate their location within the combined solutions. As we increase PEG concentration, initially we see a negligible effect on the λ-DNA diffusivity distribution (shades of blue). At 50 mg/mL, the solution is above a threshold concentration of PEG 6000 and we observe a reduction in the λ-DNA diffusivity distribution, including a sharp decrease beyond the overlap concentration for PEG 6000 at 100 mg/mL (shades of yellow-orange). The initial reduction stems from the polymer-and-salt-induced (psi or ψ) condensation by macromolecular crowding-induced depletion forces. We show the regime over which λ-DNA is condensed and decondensed along with the location of the PEG population. Distributions are derived from 10–15 runs per individual measurements and averages of several measurements.

We induced compaction of λ-DNA by macromolecular crowding using the inert, neutral flexible polymer poly(ethylene glycol) (M_n_ 6000, PEG 6000) in the presence of a divalent cation (Mg^2+^) often called polymer-and-salt-induced (psi orψ) condensation.[32] This allowed us to remove *in situ* nuclear artifacts of DNA-binding proteins (particularly histones) and the resulting ionic interactions to isolate the direct effect of condensation-induced changes on structure and mechanics that influence the fluorescence lifetime. Above a threshold concentration of PEG in the presence of divalent cations, DNA condenses due to depletion forces between PEG and DNA through the entropically-favored increase in excluded volume for PEG associated with this condensation.[33,34] We characterized the onset of DNA condensation using dynamic light scattering (DLS), where we observed a reduction in diffusivity consistent with DNA condensation for [PEG] of 50 mg/mL (Fig 2).[35] Further addition of PEG, including beyond the overlap concentration,[35] results in a greater reduction in the diffusivity that likely indicates the reduced diffusivity of multi-molecular aggregates[36] of condensed DNA from the increased solution viscosity, the production of larger multi-molecular aggregates or both (Fig 2).

**Fig 3:**
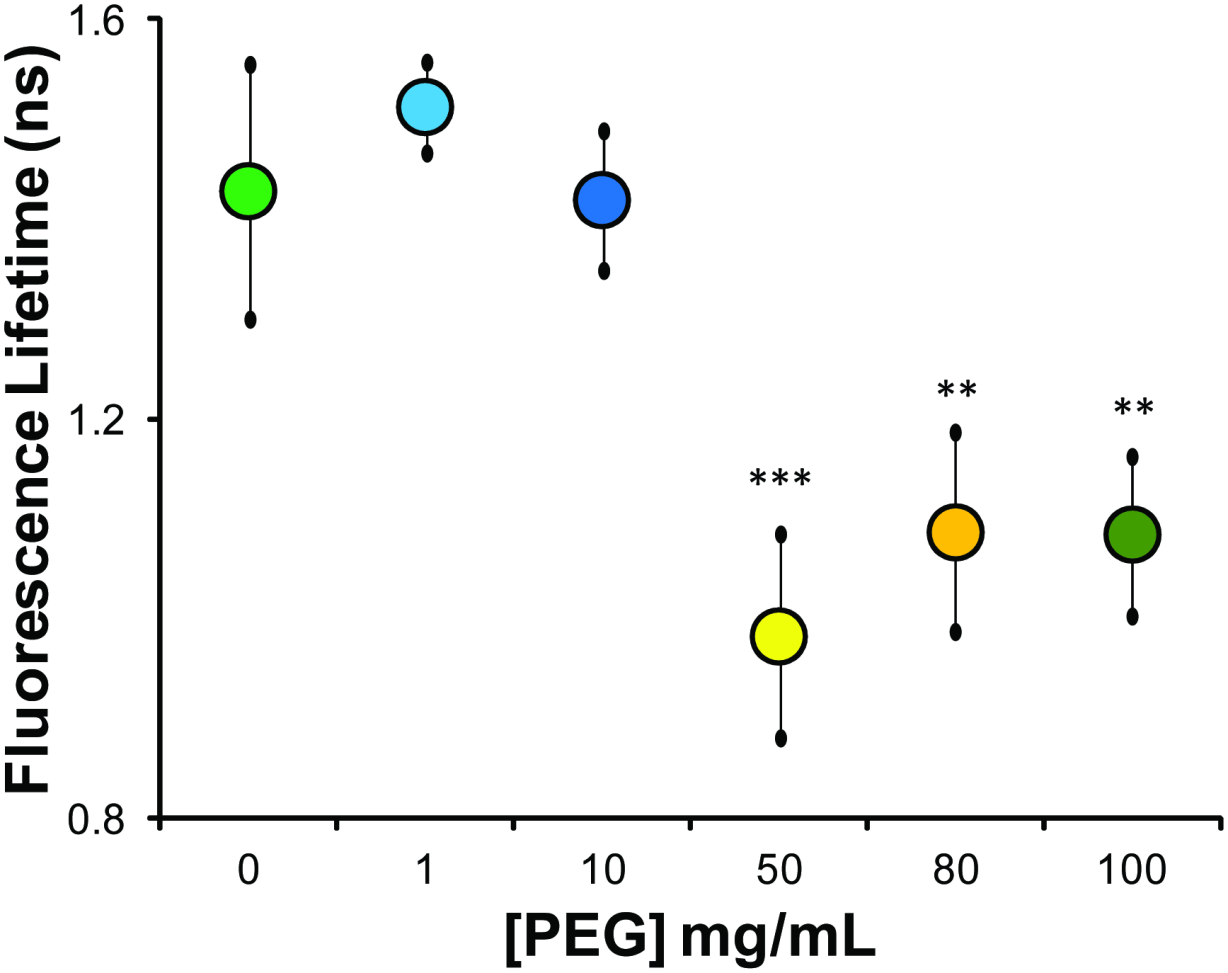
Fluorescence lifetime measurements of *in vitro* λ-DNA solutions of varying condensation state. As in the DLS experiments, we observe a dramatic reduction in the mean fluorescence lifetime above the threshold PEG 6000 concentration (~50 mg/mL; p<0.01) that is maintained at higher concentrations (shades of yellow-orange symbols). Interestingly, despite the increase in viscosity that occurs with increasing PEG concentration (including the sharp increase in trend above the overlap concentration at 100 mg/mL) we see no further statistical change in the mean fluorescence lifetime despite the dependence of the fluorescence lifetime on local viscosity. Error bars reflect standard deviation. Statistical significance based on Student’s t-test with the 0 mg/mL PEG, with **p<0.025 and ***p<0.01.

We measured the mean fluorescence lifetime associated with the PEG-induced DNA condensation and observed a significant reduction in the mean fluorescence lifetime for [PEG] of 50 mg/mL or greater (Fig 3). Note that these experiments report the fluorescence lifetime of homogeneous solutions of labeled DNA. This is in contrast to labeled DNA inside nuclei, which have an intrinsically heterogeneous distribution of lifetimes associated with the drastically larger number of possible conformations enabled by molecular crowding and specific binding proteins and complexes. As such, the requirements for statistical significance are different between *in situ* imaging and *in vitro* solution imaging (see Methods). These results are consistent with the *in situ* experiments of induced chromatin condensation in HUVEC nuclei where it resulted in a reduced mean fluorescence lifetime (Fig 1). Interestingly, following DNA condensation there was no further change in the mean fluorescence lifetime despite increasing solution viscosity associated with higher [PEG] including the sharp increase beyond overlap concentration[35] at 100 mg/mL. Thus, upon DNA condensation the fluorescence lifetime seemingly becomes largely insensitive to further changes in viscosity.

**Fig 4:**
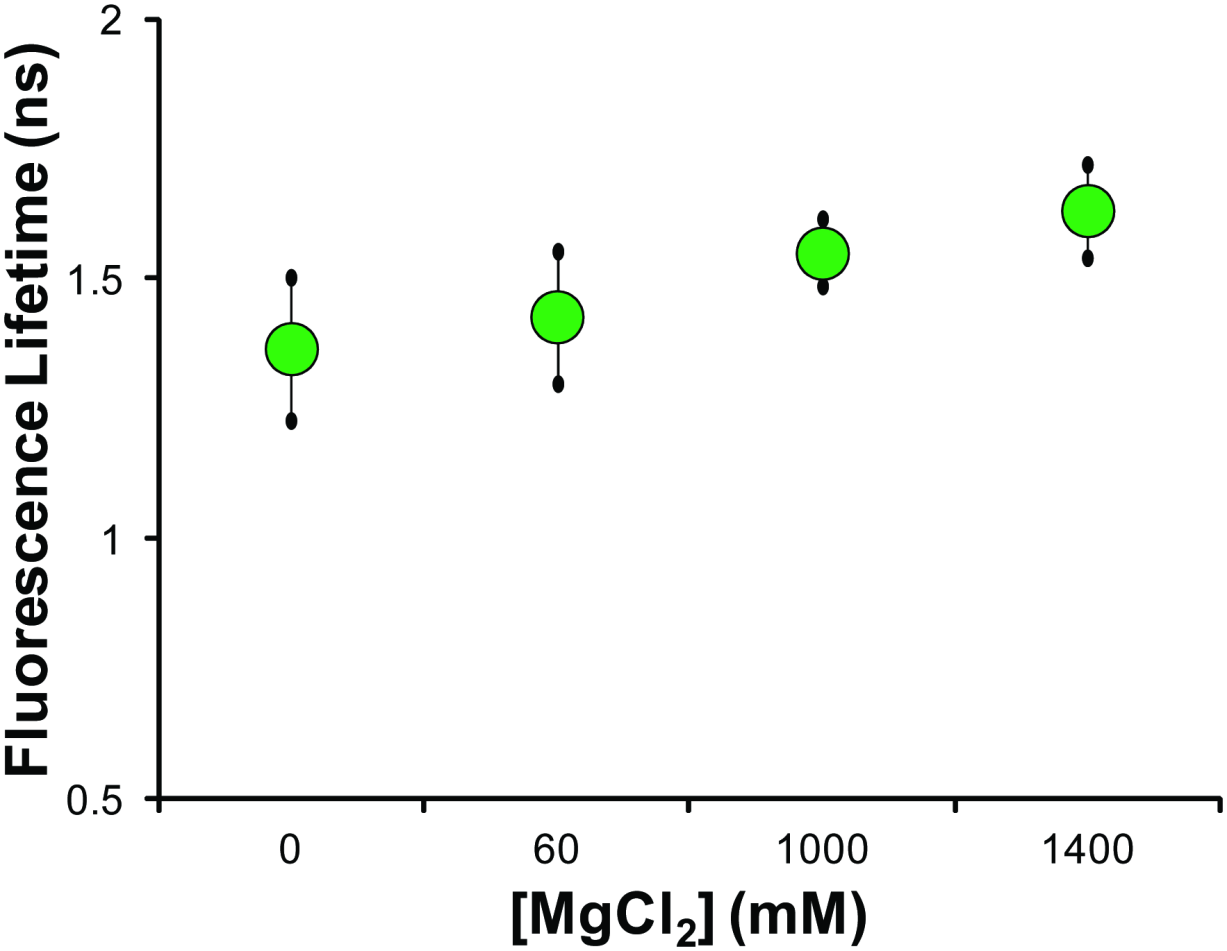
Fluorescence lifetime measurements of *in vitro* λ-DNA solutions of varying ionic strength solutions. The mean fluorescence lifetime of solutions of λ-DNA with varying concentration of MgCl_2_ shows no statistical dependence on ionic strength. Across a wide distribution of salt concentration varying over three orders of magnitude we see no statistically significant effect on the mean fluorescence lifetime, indicating it is not strongly influenced by salt concentration. Statistical comparisons made by Student’s t-test, with no statistical difference between solutions.

PEG allows for investigation of the role of DNA compaction (though only moderately reducing the dielectric constant over this range of concentration[37]). To determine impacts of ionic strength we varied solution polarity using solutions of varying [MgCl_2_] since local polarity changes in the cell likely stem predominately from ion flux. We observed no statistically significant change in the mean fluorescence lifetime with increasing MgCl_2_ concentration over a broad range spanning orders of magnitude from 0 M to more than 1 M (Fig 4). Our upper concentration of 1.4 M MgCl_2_ is orders of magnitude higher than physiological concentrations of Mg^2+^ (free Mg^2+^ at 0.5 mM and bound Mg^2+^ at 20 mM)[38] as well as the levels needed to observe dramatic chromatin reorganization (6 mM).[25] This is consistent with the relative insensitivity of the fluorescence intensity of bis-benzimide (Hoechst family) dyes with salt concentration upon binding DNA.[39]

**Fig 5:**
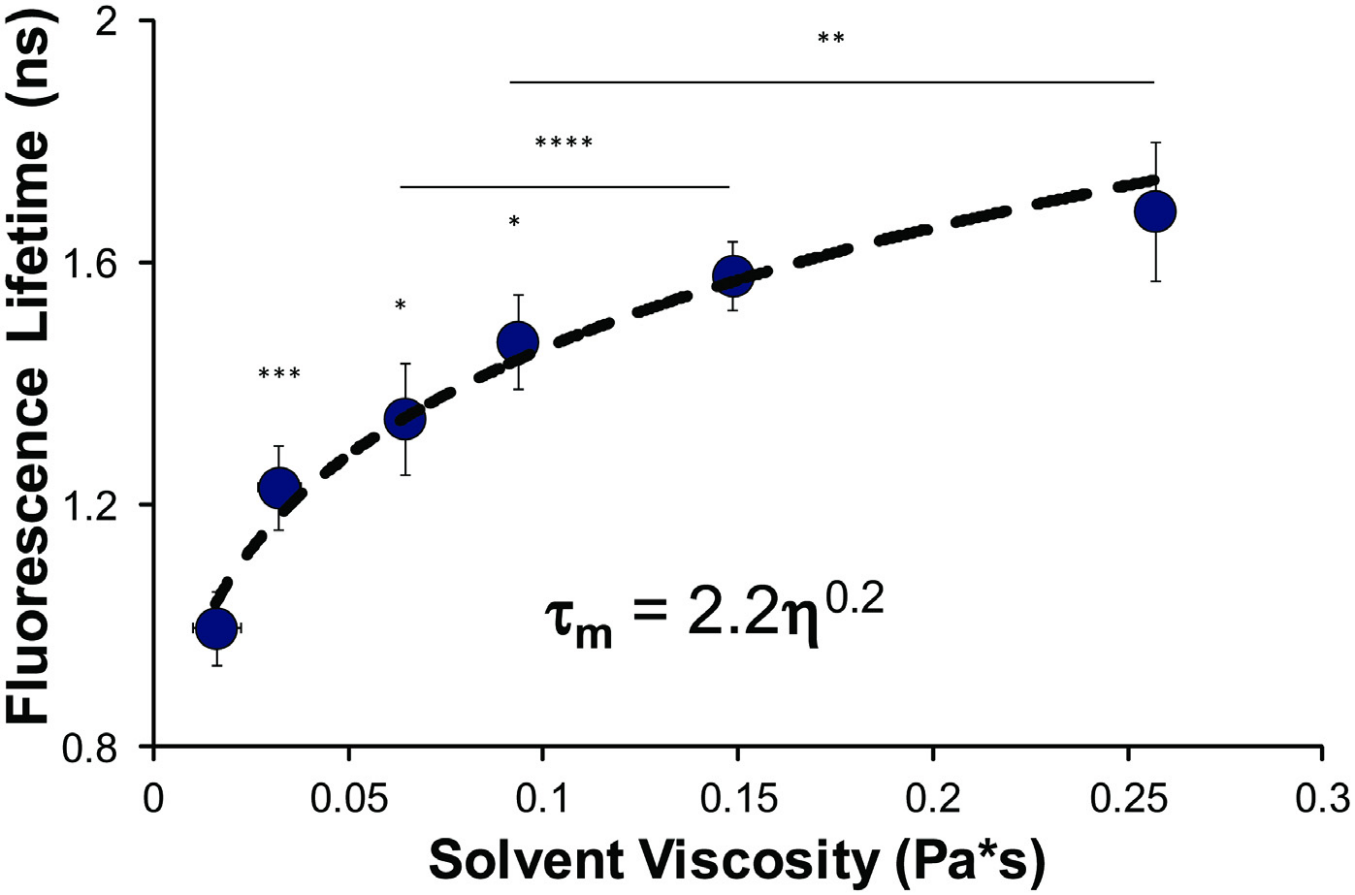
Fluorescence lifetime measurements of *in vitro* λ-DNA solutions of varying viscosity. We determine the mean fluorescence lifetime dependence of Hoechst 33342 bound to λ-DNA in solutions of varying viscosity glycerol-ethylene glycol solutions. We see a strong dependence of the mean fluorescence lifetime on viscosity over the range here. Viscosity measurements for the glycerol-ethylene solutions were determined using a Discovery Hybrid Rheometer-2. Statistical comparisons made by Student’s t-test, with *p<0.05, **p<0.025, ***p<0.01 and ****p<0.001. All statistical comparisons of the magnitudes are with the previous point unless otherwise indicated by lines. The viscosity power-law fit dependence of the mean fluorescence lifetime, based on the known phenomenological relationship between fluorescence lifetime and viscosity, was determined using ANOVA (p<<<0.001).

Using glycerol-ethylene glycol solutions of varying concentration, we controlled for media polarity changes due to the similarity of their dielectric constants (as is convention for assaying the viscosity dependence of fluorescence lifetime measurements[29]) thereby allowing us to effectively isolate the fluorescence lifetime dependence of non-compacted DNA on viscosity alone. We observed a statistically significant increase in fluorescence lifetime magnitudes with viscosity, and confirmed the significance of the power-law trend (~η^0.2^) of fluorescence lifetime with solution viscosity (Fig 5). The strong statistical significance of the power-law fit (ANOVA p<<0.001) indicates the dominant role of viscosity on the fluorescence lifetime of Hoechst 33342 bound to DNA. However, the dependence of the fluorescence lifetime on solution viscosity appeared to be less significant than the dependence on DNA condensation state. In aggregate, these experiments indicate that Hoechst 33342 bound to DNA is mostly insensitive to local solution ionic strength, but is dependent on viscosity of the solution around fluctuating DNA and strongly dependent on DNA condensation state.

### Spatially-Resolved *in situ* Chromatin Condensation State

In addition to quantifying and spatially resolving chromatin condensation state during physiological and pathological changes, the ability to investigate chromatin condensation state in the context of ongoing functional processes enables us to study the role of these deeper layers of genome function in facilitating nuclear organization. Previous work has highlighted the role of chromatin in serving to nucleate *de novo* formation of functional sites within the nucleus, thereby lending credibility to the idea that the nucleus is a self-organizing system.[3] FLIM provides the means to investigate the interrelation of chromatin structure and organization with function in the nucleus through the enhanced spatial resolution.

**Fig 6:**
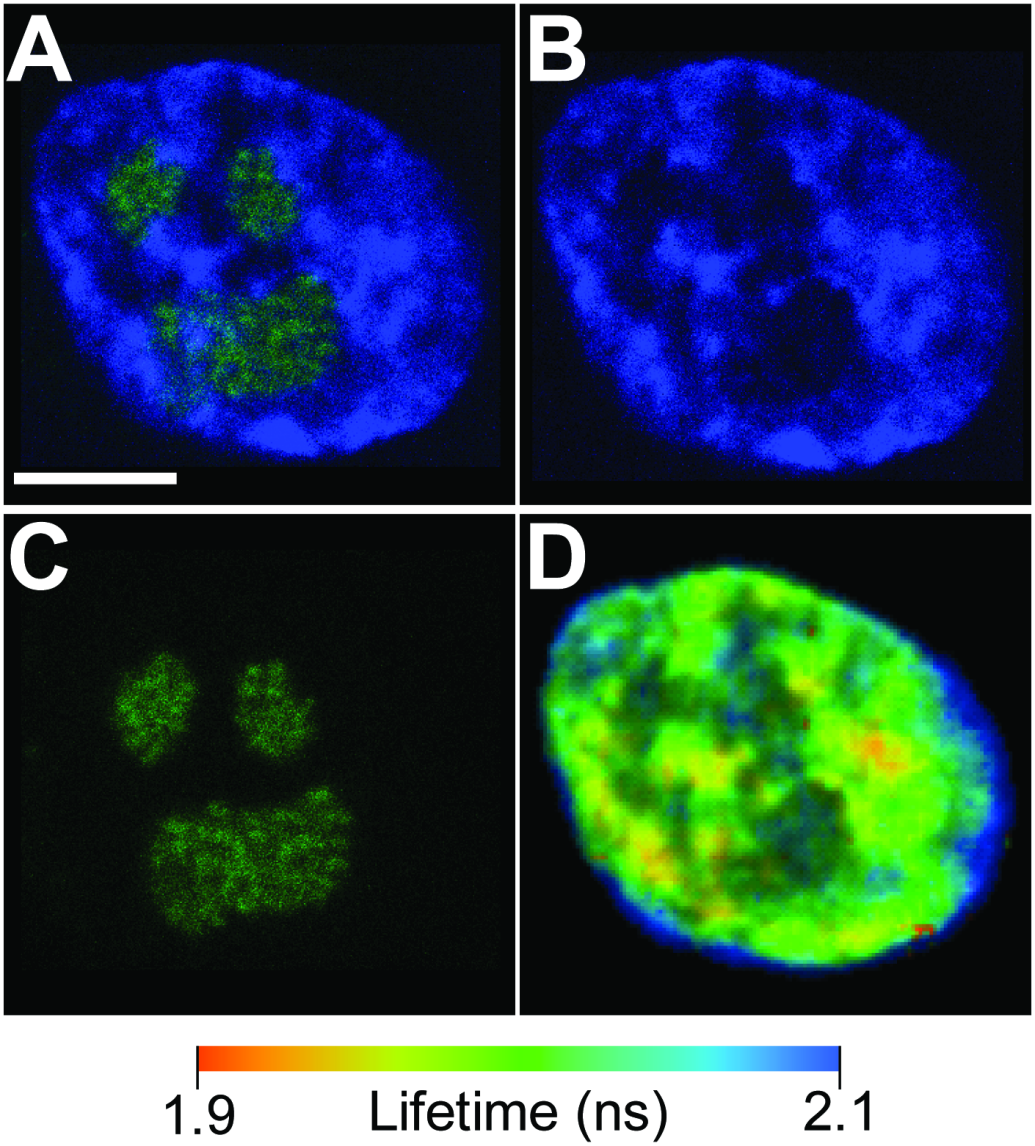
Fluorescence lifetime spatial distribution around nucleoli in endothelial cell nuclei. (A) An overlay of a confocal image of HUVECs labelled for chromatin with Hoechst 33342 (blue, B) and nucleoli with transfected nucleolar protein GFP-Fibrillarin (green, C). (D) The spatially resolved mean fluorescence lifetime of the same nucleus shows largely decondensed chromatin within the interior of the nucleolus (deep blue regions). Further, the nucleoli are bounded by condensed chromatin (yellow-orange regions) consistent with heterochromatin-bound nucleolar regions. Other condensed chromatin regions show correspondence with the brighter, more punctate regions of chromatin fluorescence (B) consistent with heterochromatin. Scale bar is 5 μm.

We transfect HUVECs with the nucleolar protein GFP-Fibrillarin as our indicator of nucleolar location since it is present at active ribosomal gene transcription centers.[38,40] The chromatin organization associated with the nucleolus, being a functional site of very low internal chromatin composition[41] as well as mostly homogenous chromatin structural state associated with decondensation for active ribosomal gene transcription on the interior that is then bounded by dense heterochromatin,[15] makes it an ideal candidate for testing the spatial resolution of FLIM for assaying chromatin condensation state. Consistent with the known organization of chromatin condensation state of nucleoli, we see the internal nucleolar regions are associated with some of the highest mean fluorescence lifetime regions of the nuclear interior in the mean fluorescence lifetime heat maps indicating highly decondensed chromatin (Fig 6). To ensure that fluorescence lifetime measurements were solely those of the Hoechst 33342, we used a tunable FLIM-specific photomultiplier tube and collected the spectra unique to each fluorophore (see methods). The wavelength of multiphoton excitation does not coincide with that of Hoechst 33342, as is evident given the low-intensity of the locations corresponding to the nucleolus in Fig 6. Further, we observe that these regions are bounded by a noticeable amount of very low mean fluorescence lifetimes associated with tightly condensed heterochromatin domains that bound the nucleolus. The slight overlap of lower mean fluorescence lifetime over a portion of the GFP-Fibrillarin signal likely results from known chromatic aberrations and z-plane differences associated with the near infrared[42] (high wavelength of 825 nm) multiphoton excitation of Hoechst 33342 utilized for FLIM relative to the visible laser (low wavelength of 488 nm) excitation of GFP.

**Fig 7:**
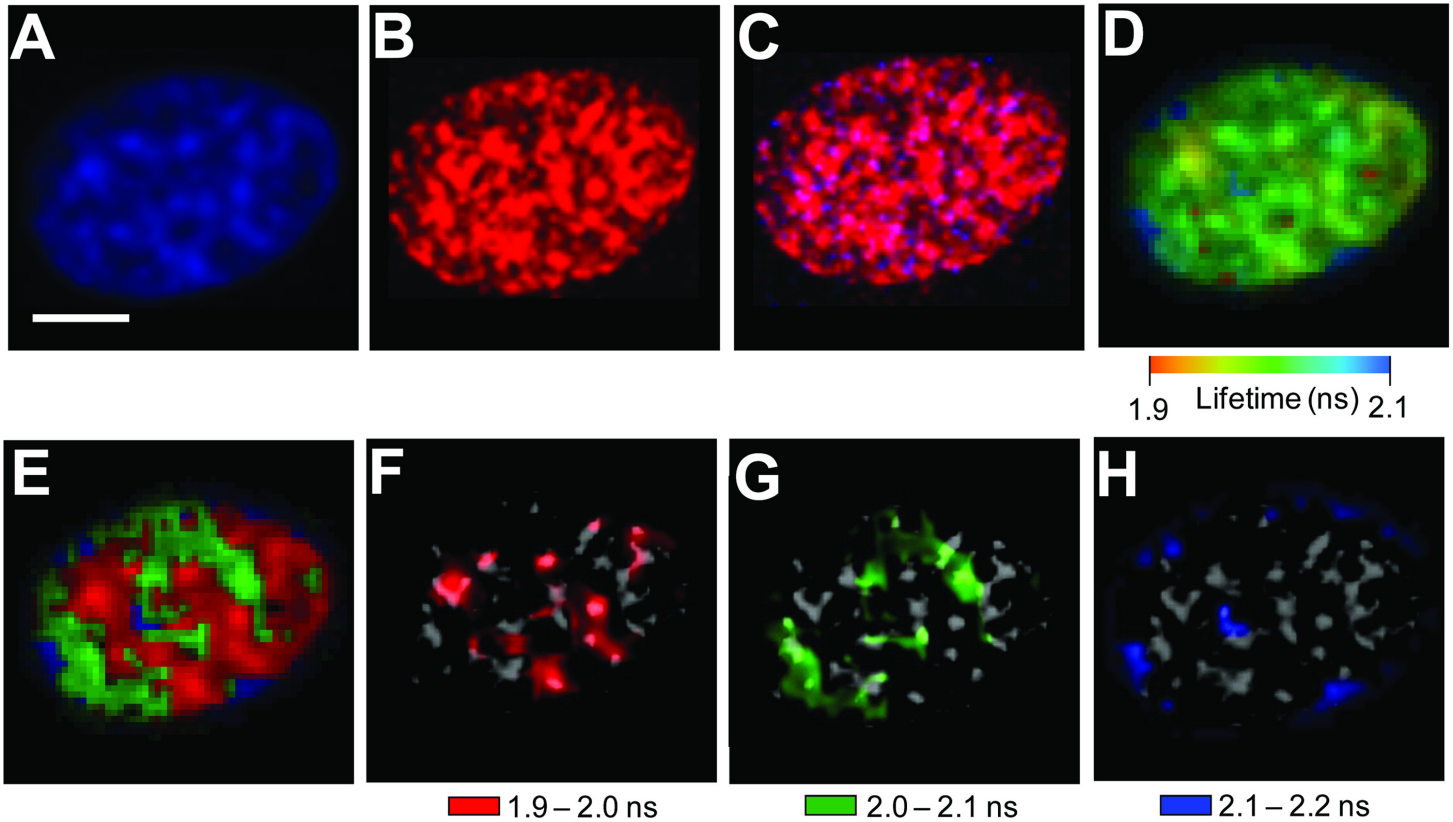
Fluorescence lifetime spatial distribution around of endothelial cells labelled for heterochromatin marker H3K9me3 with immunocytochemistry. A confocal image of a HUVEC nucleus labelled for chromatin with Hoechst 33342 (blue, A) and constitutive heterochromatin marker histone H3 tri-methylated at lysine residue 9 (H3K9me3) by immunocytochemistry (red, B) with merge (C). (D) The spatially resolved mean fluorescence lifetime of the same nucleus shows a spatially heterogeneous lifetime distribution. (E) Binning of fluorescence lifetimes rather than a continuous scale (from D) allows discrete segregation for overlay. (F) Very low lifetime regimes (red) overlaid with enhanced H3K9me3 signal (white) shows alignment of these regions. Intermediate lifetimes (green, G) show some small colocalization, but high lifetime regimes (blue, H) show no overlap with H3K9me3 staining. Scale bar is 5 μm.

We also compare the fluorescence lifetime measurement of chromatin condensation state with the constitutive heterochromatin marker H3K9me3 labeled by immunocytochemistry (Fig 7B). While the heterochromatin marker provides for a decently punctate image of heterochromatin regions, the reliance solely on the fluorescence intensity of the measurement with limited spatial resolution obfuscates clear delineation of the gradations of heterochromatin regions since it merely denotes the presence or absence of H3K9me3 (as discussed previously). Nonetheless, we qualitatively observe reduced mean fluorescence lifetimes associated with the most prominent regions of the heterochromatin label, particularly when bright regions of H3K9me immunocytochemistry signal are enhanced and overlaid with binned fluorescence lifetime data (Fig 7F–7H).

## Discussion

The quantitative visualization of chromatin structure and organization within the nucleus, including its condensation state and spatial arrangement, remains a major obstacle to understanding genome function. The capacity to spatially resolve chromatin condensation state throughout the nucleus has major bearing on understanding both the functional attributes associated with those states and the dynamics of chromatin throughout the nuclear interior. Our emerging understanding of stem cell differentiation[43] as well as cancer pathology[44] related to DNA repair[17] has highlighted the integral role of chromatin structural modifications and repositioning associated with these functional processes. Yet, questions still remain as to the causal relationship of chromatin organization with these functions including the influence of the structural state and dynamic movements. Here we aimed to address the limitation of quantification of chromatin condensation *in situ* through the development of a technique for high-throughput quantification of chromatin condensation state and it spatial arrangement in a primary human cell line using only a cell-permeable, DNA-binding fluorophore via fluorescence lifetime and its unique dependence on the mechanical-structural interrelation of chromatin. Although quantification of fluorescence intensity of Hoechst 33342-labeled chromatin has limitations, FLIM is largely independent of the intensity of the fluorophore. Studies with different DNA binding fluorophores show similarly altered fluorescence lifetime. Also, this method can use but does not require transfection, fixation, permeabilization or disruption of large scale chromatin structure to allow in large macromolecules (e.g., antibodies, hybridization partners) and provides submicron-scale resolution of DNA and chromatin condensation states down to the length scale of angstroms.

### Physical Effectors Influencing the Fluorescence Lifetime Dependence of Chromatin Condensation State *in situ* and *in vitro*

**Fig 8:**
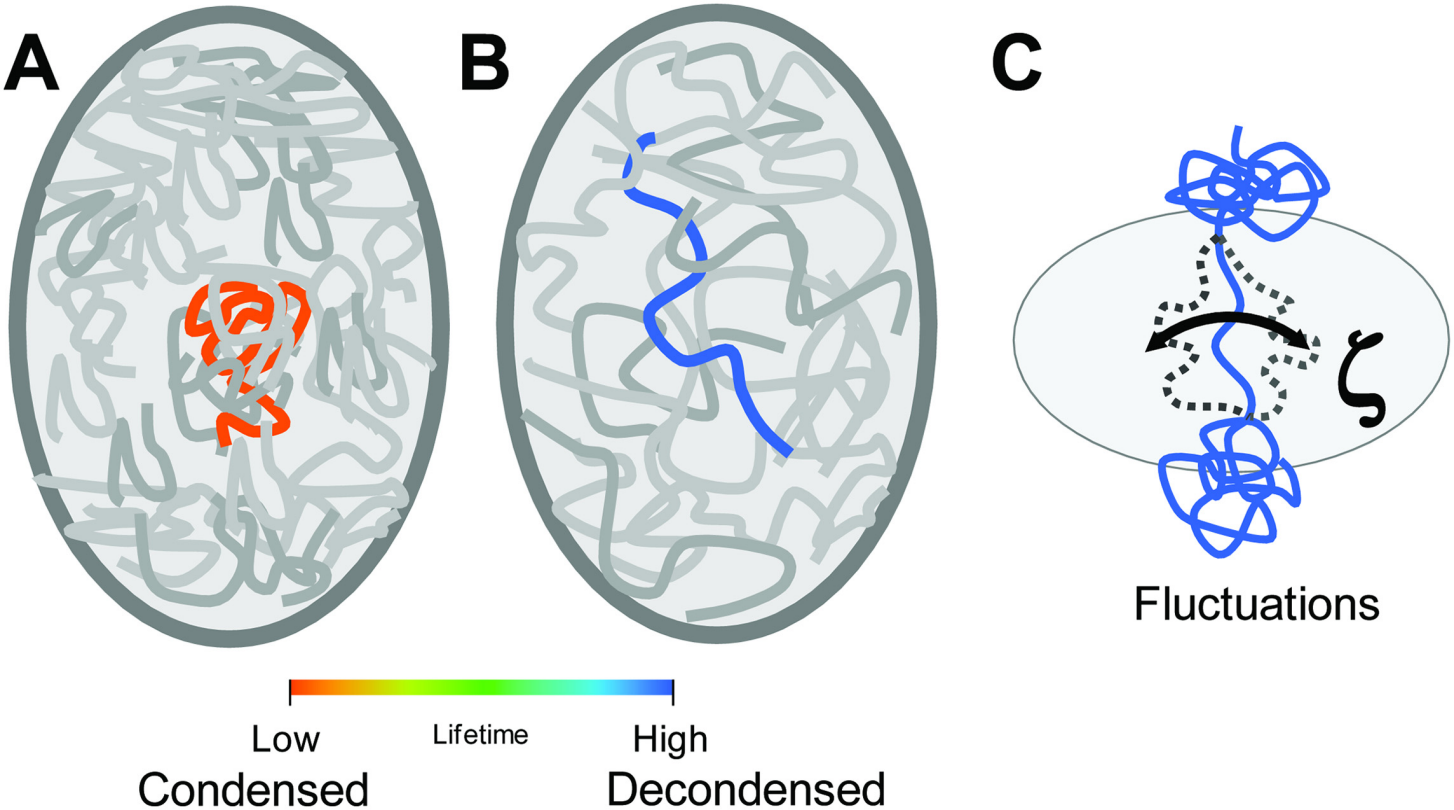
Changes in chromatin condensation state in the nuclear interior impact the local viscosity which strongly influence the fluorescence lifetime. In most cells, the nucleus has regions of highly condensed chromatin (A). While the concentration of chromatin in the nuclear interior is unchanged upon decondensation, the nuclear interior becomes more viscous due to the reduction in densely packed chromatin (B). As such, the chromatin in condensed regions has a low fluorescence lifetime (orange in A) and chromatin in decondensed regions has a high fluorescence lifetime (blue in B). (C) Decondensed chromatin undergoes fluctuations influenced by frictional drag (ζ) from the surrounding environment. Regions of higher viscosity arising from chromatin decondensation have increased mean fluorescence lifetimes.

We show the dependence of fluorescence lifetime on chromatin condensation state in primary human cell nuclei using conventional chemical treatments for global condensation state changes. Our results indicate an increase in the fluorescence lifetime corresponds to chromatin decondensation in human cell nuclei and *vice versa* (Fig 8A and 8B). Further, using macromolecular crowding-induced DNA condensation (ψ condensation) for *in vitro* solutions, we demonstrate that the fluorescence lifetime change is dependent on the altered structural state associated with condensation independent of the presence of binding proteins. We highlight that the physical parameter impacting the fluorescence lifetime change upon condensation is the stark transition in the sensitivity to viscosity associated with these two states of DNA condensation. Decondensed λ-DNA, being a largely flexible polymer (L_C_ ~ 16.2 μm >> L_P_ ~ 0.05 μm[45]), maintains a compliant mechanical state undergoing large length scale (relative to the Hoechst 33342 minor groove-binding site of ~2 nm[38,46]) Brownian polymeric fluctuations that render the fluorescence lifetime sensitive to local solution viscosity (Fig 8C). After condensation, these large length scale λ-DNA fluctuations are reduced, diminishing the sensitivity to the viscosity and decreasing the local solution viscosity due to the simultaneous condensation of other λ-DNA molecules. Within the context of the nucleus (Fig 8A and 8B), chromatin condensation similarly corresponds to a less viscous environment relative to decondensed chromatin. This “switch” sensitivity upon condensation renders the fluorescence lifetime sensitive to the condensation state and intermediate conformations via the fiber compliance and its dependence on viscosity when not condensed. Previous works have similarly demonstrated chromatin decondensation results in more viscous and deformable cell nuclei.[26,27]

The lack of a strong influence of [MgCl_2_] on the fluorescence lifetime is somewhat surprising given the general association of fluorescence lifetime of molecules in solution on ionic strength through polarity.[29] However, we suggest that the minor-groove binding of Hoechst 33342 to DNA[46] makes the fluorophore sensitivity to the media ionic strength relatively negligible likely due to the presence of interactions with the base pairs themselves (where hydrogen bonds[47] and possibly aromatic ring interactions may occur). This idea is supported by the reduced sensitivity of bis-benzimide (Hoechst family) dyes to pH (over a broad range) upon binding to DNA as well as the relative insensitivity of the fluorescence to [MgCl_2_] and [NaCl] over broad ranges[48] in the bound state. Thus, upon the binding of Hoechst 33342 to chromatin, the chromatin structural state and its resulting effect on local viscosity and as well as the sensitivity of its fluctuations to viscosity provides the dominating environmental characteristic influencing fluorescence lifetime. This is consistent with the known effect of increased viscosity on increasing the fluorescence lifetime of the Hoechst family of dyes, including a dramatic increase upon binding DNA.[29]

Previous work has demonstrated that the viscosity dependence of the fluorescence lifetime can be considerable depending on the internal flexibility of the fluorophore structure, where those that more freely undergo internal rotations (often termed “molecular rotors”) have strong fluorescence lifetime dependence on viscosity.[29] In this case, the binding of Hoechst 33342 to DNA renders the fluorescence lifetime susceptible to both the structural state of the DNA and the viscosity of the surrounding medium, which influences the ability of DNA to reptate or fluctuate. Thus, given the length scale dependence of fluorescence lifetime being on the order of ~0.1–10 nm and the absence of a change in the fluorescence lifetime over wide ranges of ionic strength, the condensation state of DNA in the context of Brownian polymeric fluctuations and the associated viscous drag becomes paramount to the fluorescence lifetime dependence (Fig 8C). Since these Brownian fluctuations depend on both the structural state of the polymer itself and the resistance in the surrounding medium (viscosity), the “switch” sensitivity of the fluorescence lifetime enables us to quantify chromatin condensation state through the reduced mechanical flexibility and concomitant reduced viscosity associated with increasing condensation. This dependence of the fluorescence lifetime on chromatin condensation state through mechanics provides a high-throughput technique for quantifying and spatially resolving condensation state throughout the nucleus.

### FLIM to Measure Chromatin Structure and Mechanics *in situ*

The utility of the fluorescence lifetime measurement of chromatin condensation lies in its ability to be done *in situ* and with spatial resolution for a variety of cell nuclei under varying physiological and pathological conditions. Where previous methods have failed to provide the proper resolution or be utilized for primary cell lines, the fluorescence lifetime proves capable of surmounting these limitations through its dependence on mechanical chromatin compliance as related to its condensation state. We show the utility of these measurements for spatially resolved chromatin condensation at distinct functional sites. Spatially resolving the chromatin structure within and around nucleoli highlights the capability of FLIM for determining the role of chromatin condensation in the context of functional organization. While ribosomal biogenesis in the nucleolus is a continuous process in interphase nuclei, FLIM can similarly be utilized to capture snapshots of chromatin condensation with respect to the progression of other functional processes. This affords the opportunity to monitor the role of chromatin hierarchical organization (higher-order structural state and spatial arrangement) in the context of genome function during physiological changes.

While particle tracking and other diffusivity measurements boast the benefit of additional biological information associated with the driving forces of chromatin fluctuations (and, thus, motor activity),[16] FLIM allows for mapping of the spatial distribution of chromatin condensation states as well as high-throughput capacity relative to other mechanical methods. Particle tracking experiments yield results of many particles, but often only capture a limited number of cells per experiment due to transfection limitations[28,49] and require long duration experiments to capture evolving phenomena of biological processes.[16,50] Similarly, faster diffusivity measurements of free fluorescent particles in the nucleus may provide insight into other length scale and time scale dependent behavior,[22–24] but validating the precise phenomena responsible for the underlying changes (particularly at precise spatial locations) is difficult and such measurements generally require cell transfection as well. By contrast, FLIM measurements allow up to 30–80 nuclei in a single field of view (pending cell type and density) to be sampled in <10 min (in some cases 1 min) without the need for transfection. This, coupled with the capacity to resolve rapid condensation state changes on these shorter time scales and within the context of other labeled functional sites, provides an ideal technique for understanding chromatin structural state changes and positioning effects as they are directly related to function.

Using FLIM to quantify changes in chromatin condensation state in cells under various physiological or pathological conditions provides the means to investigate the hierarchical layers of chromatin organization in this context *in situ*. Recent efforts aimed at understanding the physiology of cell lineage determination during stem cell differentiation hinge in part on the ability to determine how the cell nucleus organizes the genome during these changes, including epigenetic modifications for chromatin remodeling and altered chromatin association with the nuclear periphery.[43] FLIM proves capable of simultaneously monitoring both as well as the temporal evolution. This would also enable better understanding of cellular reprogramming for potential therapies, where the same phenomena are at play.[51] Another potential application is the identification[52] or investigation of[53] cancerous cell types, which are hallmarked by aberrant nuclear organization and dysregulation of the genome. Determining whether structural defects in chromatin organization cause the observed phenotypes or if they are symptomatic of altered gene expression from local changes at a particular gene is current obstacle in cancer pathology for which FLIM would be useful. These future applications of FLIM for assaying hierarchical chromatin organization in human cell nuclei highlight the utility of this high-throughput mechanical technique that provides a promising tool for investigating these deeper layers of genome function.

## Materials and Methods

### Cell Culture and Transfection

For our cell experiments, we used human umbilical vein endothelial cells (HUV-EC-C [HUVEC], ATCC CRL-1730^™^, Manassas, VA) that were cultured in endothelial basal media with endothelial growth supplements (Lonza, Hopkinton, MA). This particular HUVEC line is cultured minimally commercially to maintain consistency; these HUVECs are a non-continuous line being pooled from many individuals but are not immortalized and only survive for 10 passages in culture. Cells were used between passage 2–8. Cells were passaged onto glass slides (VWR International, Radnor, PA) in 35 mm tissue culture dishes (Corning Inc., Corning, NY). For transfection experiments, cells were transfected using the endothelial cell-specific Lipofectin transfection reagent (Life Technologies, Grand Island, NY) in Opti-MEM I Reduced Serum Medium (Life Technologies, Grand Island, NY). For these experiments we used rDNA of GFP-Fibrillarin (kind gift from D. Discher, University of Pennsylvania). Cells were incubated for transfection for five hours, at which time we changed to normal growth media and incubated the cells for an additional 24–72 hours prior to experiments to allow for adequate expression and proliferation to confluency.

### Drug Treatments

Chromatin condensation experiments were run using either trichostatin A (TSA, 200 ng/mL for 24 hours) for decondensation or sodium azide (NaN_3_, 10 mM) and 2-deoxyglucose (2-DG, 50 mM) for one hour for ATP depletion-induced chromatin condensation.

### Cell Fixation and Staining

Cell fixation was done using 3.7% formaldehyde in phosphate buffered saline (PBS). Cell permeabilization was done using 0.2% Triton X-100 in PBS. While this chemical fixation may somewhat alter the local chemical environment as a result of crosslinking, previous studies have demonstrated that DNA and nucleosomes in cells fixed via formaldehyde maintain considerable mobility[31] indicating local chromatin structures are largely preserved at these small length scales. For fluorescence lifetime imaging microscopy (FLIM) experiments, cells were stained with 5 μg/mL Hoechst 33342 (Life Technologies, Grand Island, NY) or PicoGreen in PBS. For immunocytochemistry labeling experiments, cells were blocked with 0.2% bovine serum albumin (BSA, Sigma Aldrich, St. Louis, MO) in PBS. We labeled the constitutive heterochromatin marker Histone H3K9me3 with the primary rabbit polyclonal antibody (ab8898, Abcam, Cambridge, England) and a secondary donkey anti-rabbit antibody (sc-362291, Santa Cruz Biotechnology, Dallas, TX).

### Preparation of DNA *in vitro* Solutions

For the DNA *in vitro* solution experiments, double-stranded λ-DNA was obtained from New England Biolabs (Ipswich, MA). All solutions were sterile-filtered. The base buffer was 20 μg/mL of λ-DNA with Hoechst 33342 in 60 mM MgCl_2_ in 10 mM Tris-HCl. For the ψ-condensation (Polymer-and-Salt-Induced-condensation) experiments, poly(ethylene glycol) 6000 (PEG 6000) was used (M_n_ 6,000, Sigma-Aldrich, St. Louis, MO) and serially diluted to the proper solution concentration with the base buffer. Dynamic light scattering (DLS) experiments of DNA-PEG 6000 solutions were run using a Malvern Zetasizer Nano ZSP (Malvern, Worcestershire, UK). The fluorescence lifetime dependence experiments with varying ionic strength were done using serial dilutions of MgCl_2_ in 10 mM Tris-HCl for the indicated concentrations. The fluorescence lifetime viscosity dependence experiments were done using serial dilutions of glycerol-ethylene glycol. Viscosity measurements of glycerol-ethylene glycol solutions were made using Discovery Hybrid Rheometer-2 (TA Instruments, New Castle, DE) using a shear rate sweep of 0.1–10,000 s^-1^.

### Fluorescence Lifetime Imaging Microscopy

Our FLIM setup utilized a Leica TCS SP5 inverted laser scanning confocal microscope and a 100x (1.4 NA) oil immersion objective. For excitation in the FLIM experiments, a Ti:sapphire mode-locked, pulsed infrared laser (Chameleon, Coherent) system was utilized as the multiphoton excitation source (1 W, average) tuned to 825 nm (Hoechst 33342) or 925 nm (PicoGreen) with pulse-widths of <140 fs delivered at 90 MHz. For emission, a FLIM-specific photomultiplier tube (PMT) was used and collected the spectra from 404–536 nm (Hoechst 33342) or 500–600 nm (PicoGreen). Fluorescence lifetime data was acquired and analyzed using previously published methods[16,54] with a suite of software from Becker & Hickl SPC-830 for time-correlated single photon counting (TCSPC) with 10 ps resolution along with 220 time channels and a 10.8 ns measurement window. Scans lasted at least 180 s for images of 256 pixels × 256 pixels.

The decay rate of the fluorescence lifetime can be modeled as a summation of exponential decays (Eq 1), where τ_n_ and a_n_ are the lifetime and normalized amplitude of the n^th^ exponential decay, respectively. I(t) is the number of photons detected per unit time, t, and I_0_ is the offset for the background. The mean fluorescence lifetime is defined as shown in Eq 2.

I(t)= I0+∑nane−tτn (1)

$$\tau_{m}=\frac{\sum_{n}a_{n}\tau_{n}}{\sum_{n}a_{n}}$$ (2)

The heat maps of the fluorescence lifetimes were created in Becker & Hickl SPCImage software along with the data analysis. For cell experiments, we segmented the nuclei in each field of view to isolate only nuclear pixel signal for data analysis using MATLAB. We analyzed the fluorescence lifetime fits using a χ^2^ test, with Hoechst 33342 best modeled by a double exponential decay while PicoGreen was modeled best as a single exponential decay.

### Statistics

Magnitudes of the mean fluorescence lifetimes were statistically compared using Student’s t-test. Fits of the fluorescence lifetime exponential decay were verified using a χ^2^ test. The fit of the fluorescence lifetime dependence of Hoechst 33342 bound to λ-DNA with varying viscosity was tested using ANOVA (p<<0.001). For nuclear chromatin condensation state experiments using FLIM, the mean fluorescence lifetimes and associated standard deviations in the figures reflect those for segmented nuclei, with the final magnitudes resulting from pixel-to-pixel averaging within segmented nuclei across multiple nuclei per field of view and multiple fields of view per treatment condition. By contrast, in the FLIM measurements of *in vitro* λ-DNA solutions, the mean fluorescence lifetimes and associated standard deviations in the figures reflect the averaging of pixel-to-pixel lifetime values in a field of view across multiple fields of view.

## Supporting Information

S1 File Fluorescence lifetime measurements of chromatin condensation state in fixed and live human umbilical vein endothelial cell nuclei. **Fig A.** Fluorescence lifetime heat maps of chromatin is measured in endothelial cell nuclei labeled with Hoechst 33342. Fixed samples, similar to those used throughout a majority of the results, show a spatial distribution of lifetimes. Live cells also show a spatial distribution of lifetimes in the same regime. **Fig B.** The mean fluorescence lifetime of segmented nuclei for the various treatment conditions was calculated using Eq 2. Samples were statistically similar with one another. Live cells showed greater variation likely from exchange of dye between the nucleus and media as well as cell motility.
(DOCX)

S2 File Distributions of fluorescence lifetime in nuclei of cells with different treatments. **Fig A.** Histograms of lifetimes are shown for each condition to highlight the spatial heterogeneity for each sample. NaN_3_+2-DG treatment shows a significant contribution from very low lifetimes (0.6 ns and 1.3). This reduces the mean fluorescence lifetime (dashed line). In contrast, the mean fluorescence lifetime of TSA treated nuclei is increased with a smaller variance since there is little contribution from very low lifetimes. **Fig B.** The mean fluorescence lifetime of segmented nuclei for the various treatment conditions was calculated using Eq 2. Treatment with NaN_3_+2-DG resulted in a strong reduction in the mean fluorescence lifetime relative to untreated controls. By contrast, TSA treatment resulted in a dramatic increase in the mean fluorescence lifetime relative to untreated controls as well as a large reduction in the variance which indicated an increase in chromatin condensation state homogeneity throughout the cell nucleus. Error bars indicate standard deviation of pixel-to-pixel mean fluorescence lifetime differences of segmented nuclei in fields of view across multiple fields of view under each treatment condition.
(DOCX)

S3 File Fluorescence lifetime measurements of chromatin condensation state in human umbilical vein endothelial cell nuclei with PicoGreen. **Fig A.** Fluorescence intensity confocal images and mean fluorescence lifetime heat maps of chromatin is measured in endothelial cell nuclei labeled with PicoGreen. Cells are treated with TSA for chromatin decondensation. Altered fluorescence intensity with treatments show differential chromatin condensation state, with more intense fluorescence arising from highly concentrated condensed chromatin. Mean fluorescence lifetime heat maps similarly indicate spatial arrangement of local fluorophore environments for labeled chromatin consistent with varying chromatin condensation state. Treatment with TSA resulted in a significant reduction in punctate regions and longer mean fluorescence lifetime relative to untreated controls. **Fig B.** The mean fluorescence lifetime of segmented nuclei for the various treatment conditions was calculated using Eq 2. Treatment with TSA treatment resulted in a dramatic increase in the mean fluorescence lifetime relative to untreated controls as well as a large reduction in the variance which indicated an increase in chromatin condensation state homogeneity throughout the cell nucleus. Error bars indicate standard deviation of pixel-to-pixel mean fluorescence lifetime differences of segmented nuclei in fields of view across multiple fields of view under each treatment condition. Standard deviation was used in place of standard error of the mean to emphasize the reduction in the fluorescence lifetime variance from chromatin decondensation from TSA treatment.
(DOCX)

## References

[1] Bintu L, Ishibashi T, Dangkulwanich M, Wu YY, Lubkowska L, Kashlev M, et al (2012) Nucleosomal Elements that Control the Topography of the Barrier to Transcription. Cell 151: 738–749. 10.1016/j.cell.2012.10.009 23141536 PMC3508686

[2] Martin RM, Cardoso MC (2010) Chromatin condensation modulates access and binding of nuclear proteins. FASEB J 24: 1066–1072. 10.1096/fj.08-128959 19897663 PMC2845425

[3] Misteli T (2007) Beyond the sequence: Cellular organization of genome function. Cell 128: 787–800. 17320514 10.1016/j.cell.2007.01.028

[4] Heitz E (1928) Das Heterochromatin der Moose. Jahrb Wiss Botanik 69: 762–818.

[5] Fedorova E, Zink D (2008) Nuclear architecture and gene regulation. Biochimica Et Biophysica Acta-Molecular Cell Research 1783: 2174–2184. 10.1016/j.bbamcr.2008.07.018 18718493

[6] Dahl KN, Booth-Gauthier EA, Ladoux B (2010) In the middle of it all: mutual mechanical regulation between the nucleus and the cytoskeleton. J Biomech 43: 2–8. 10.1016/j.jbiomech.2009.09.002 19804886

[7] Woodcock CL, Ghosh RP (2010) Chromatin Higher-order Structure and Dynamics. Cold Spring Harbor Perspectives in Biology 2. 10.1101/cshperspect.a000596 PMC2857170 20452954

[8] Wang YJ, Maharana S, Wang MD, Shivashankar GV (2014) Super-resolution microscopy reveals decondensed chromatin structure at transcription sites. Scientific Reports 4. 10.1038/srep04477 PMC3966049 24667378

[9] Burd CJ, Ward JM, Crusselle-Davis VJ, Kissling GE, Phadke D, Shah RR, et al (2012) Analysis of Chromatin Dynamics during Glucocorticoid Receptor Activation. Molecular and Cellular Biology 32: 1805–1817. 10.1128/MCB.06206-11 22451486 PMC3347412

[10] Ram O, Goren A, Amit I, Shoresh N, Yosef N, Ernst J, et al (2011) Combinatorial Patterning of Chromatin Regulators Uncovered by Genome-wide Location Analysis in Human Cells. Cell 147: 1628–1639. 10.1016/j.cell.2011.09.057 22196736 PMC3312319

[11] Riddle NC, Minoda A, Kharchenko PV, Alekseyenko AA, Schwartz YB, Tolstorukov MY, et al (2011) Plasticity in patterns of histone modifications and chromosomal proteins in Drosophila heterochromatin. Genome Research 21: 147–163. 10.1101/gr.110098.110 21177972 PMC3032919

[12] Geyer PK, Vitalini MW, Wallrath LL (2011) Nuclear organization: taking a position on gene expression. Curr Opin Cell Biol 23: 354–359. 10.1016/j.ceb.2011.03.002 21450447 PMC4160055

[13] Fedorova E, Zink D (2009) Nuclear genome organization: common themes and individual patterns. Curr Opin Genet Dev 19: 166–171. 10.1016/j.gde.2009.02.003 19321336

[14] Meldi L, Brickner JH (2011) Compartmentalization of the nucleus. Trends Cell Biol 21: 701–708. 10.1016/j.tcb.2011.08.001 21900010 PMC3970429

[15] van Koningsbruggen S, Gierlinski M, Schofield P, Martin D, Barton GJ, Ariyurek Y, et al (2010) High-Resolution Whole-Genome Sequencing Reveals That Specific Chromatin Domains from Most Human Chromosomes Associate with Nucleoli. Mol Biol Cell 21: 3735–3748. 10.1091/mbc.E10-06-0508 20826608 PMC2965689

[16] Spagnol ST, Dahl KN (2014) Active cytoskeletal force and chromatin condensation independently modulate intranuclear network fluctuations. Integrative Biology 6: 523–531. 10.1039/c3ib40226f 24619297

[17] Dion V, Gasser SM (2013) Chromatin Movement in the Maintenance of Genome Stability. Cell 152: 1355–1364. 10.1016/j.cell.2013.02.010 23498942

[18] Mora-Bermudez F, Ellenberg J (2007) Measuring structural dynamics of chromosomes in living cells by fluorescence microscopy. Methods 41: 158–167. 17189858 10.1016/j.ymeth.2006.07.035

[19] Seidal T, Balaton AJ, Battifora H (2001) Interpretation and quantification of immunostains. American Journal of Surgical Pathology 25: 1204–1207. 11688582 10.1097/00000478-200109000-00013

[20] Iyer KV, Pulford S, Mogilner A, Shivashankar GV (2012) Mechanical Activation of Cells Induces Chromatin Remodeling Preceding MKL Nuclear Transport. Biophys J 103: 1416–1428. 10.1016/j.bpj.2012.08.041 23062334 PMC3471483

[21] Lleres D, James J, Swift S, Norman DG, Lamond AI (2009) Quantitative analysis of chromatin compaction in living cells using FLIM-FRET. Journal of Cell Biology 187: 481–496. 10.1083/jcb.200907029 19948497 PMC2779238

[22] Hinde E, Cardarelli F, Digman MA, Gratton E (2010) In vivo pair correlation analysis of EGFP intranuclear diffusion reveals DNA-dependent molecular flow. Proc Natl Acad Sci U S A 107: 16560–16565. 10.1073/pnas.1006731107 20823232 PMC2944750

[23] Hinde E, Cardarelli F, Digman MA, Kershner A, Kimble J, Gratton E (2011) The impact of mitotic versus interphase chromatin architecture on the molecular flow of EGFP by pair correlation analysis. Biophys J 100: 1829–1836. 10.1016/j.bpj.2011.02.024 21463597 PMC3072664

[24] Capoulade J, Wachsmuth M, Hufnagel L, Knop M (2011) Quantitative fluorescence imaging of protein diffusion and interaction in living cells. Nature Biotechnology 29: 835–839. 10.1038/nbt.1928 21822256

[25] Dahl KN, Engler AJ, Pajerowski JD, Discher DE (2005) Power-law rheology of isolated nuclei with deformation mapping of nuclear substructures. Biophys J 89: 2855–2864. 16055543 10.1529/biophysj.105.062554 PMC1366783

[26] Pajerowski JD, Dahl KN, Zhong FL, Sammak PJ, Discher DE (2007) Physical plasticity of the nucleus in stem cell differentiation. Proc Natl Acad Sci U S A 104: 15619–15624. 17893336 10.1073/pnas.0702576104 PMC2000408

[27] Chalut KJ, Hopfler M, Lautenschlager F, Boyde L, Chan CJ, Ekpenyong A, et al (2012) Chromatin Decondensation and Nuclear Softening Accompany Nanog Downregulation in Embryonic Stem Cells. Biophys J 103: 2060–2070. 10.1016/j.bpj.2012.10.015 23200040 PMC3512036

[28] Zidovska A, Weitz DA, Mitchison TJ (2013) Micron-scale coherence in interphase chromatin dynamics. Proc Natl Acad Sci U S A 110: 15555–15560. 10.1073/pnas.1220313110 24019504 PMC3785772

[29] Berezin MY, Achilefu S (2010) Fluorescence Lifetime Measurements and Biological Imaging. Chemical Reviews 110: 2641–2684. 10.1021/cr900343z 20356094 PMC2924670

[30] Bacskai BJ, Skoch J, Hickey GA, Allen R, Hyman BT (2003) Fluorescence resonance energy transfer determinations using multiphoton fluorescence lifetime imaging microscopy to characterize amyloid-beta plaques. Journal of Biomedical Optics 8: 368–375. 12880341 10.1117/1.1584442

[31] Hihara S, Pack CG, Kaizu K, Tani T, Hanafusa T, Nozaki T, et al (2012) Local nucleosome dynamics facilitate chromatin accessibility in living mammalian cells. Cell Reports 2: 1645–1656. 10.1016/j.celrep.2012.11.008 23246002

[32] Bloomfield VA (1997) DNA condensation by multivalent cations. Biopolymers 44: 269–282. 9591479 10.1002/(SICI)1097-0282(1997)44:3<269::AID-BIP6>3.0.CO;2-T

[33] Kleideiter G, Nordmeier E (1999) Poly(ethylene glycol)-induced DNA condensation in aqueous/methanol containing low-molecular-weight electrolyte solutions Part II. Comparison between experiment and theory. Polymer 40: 4025–4033.

[34] Kleideiter G, Nordmeier E (1999) Poly(ethylene glycol)-induced DNA condensation in aqueous/methanol containing low-molecular-weight electrolyte solutions Part I. Theoretical considerations. Polymer 40: 4013–4023.

[35] Hirano K, Ichikawa M, Ishido T, Ishikawa M, Baba Y, Yoshikawa K (2012) How environmental solution conditions determine the compaction velocity of single DNA molecules. Nucleic Acids Research 40: 284–289. 10.1093/nar/gkr712 21896618 PMC3245929

[36] Hud NV, Vilfan ID (2005) Toroidal DNA condensates: Unraveling the fine structure and the role of nucleation in determining size. Annual Review of Biophysics and Biomolecular Structure 34: 295–318. 15869392 10.1146/annurev.biophys.34.040204.144500

[37] Arnold K, Herrmann A, Pratsch L, Gawrisch K (1985) The Dielectric-Properties of Aqueous-Solutions of Poly(Ethylene Glycol) and Their Influence on Membrane-Structure. Biochimica Et Biophysica Acta 815: 515–518. 3995041 10.1016/0005-2736(85)90381-5

[38] Alberts B, Wilson JH, Hunt T (2008) Molecular biology of the cell. New York: Garland Science 1 v. (various pagings) p.

[39] Labarca C, Paigen K (1980) Simple, Rapid, and Sensitive DNA Assay Procedure. Analytical Biochemistry 102: 344–352. 6158890 10.1016/0003-2697(80)90165-7

[40] Martin C, Chen S, Maya-Mendoza A, Lovric J, Sims PF, Jackson DA (2009) Lamin B1 maintains the functional plasticity of nucleoli. J Cell Sci 122: 1551–1562. 10.1242/jcs.046284 19383719 PMC2722682

[41] Pliss A, Kuzmin AN, Kachynski AV, Prasad PN (2010) Nonlinear Optical Imaging and Raman Microspectrometry of the Cell Nucleus throughout the Cell Cycle. Biophys J 99: 3483–3491. 10.1016/j.bpj.2010.06.069 21081098 PMC2980749

[42] Streit JK, Bachilo SM, Weisman RB (2013) Chromatic aberration short-wave infrared spectroscopy: nanoparticle spectra without a spectrometer. Anal Chem 85: 1337–1341. 10.1021/ac303713z 23286305

[43] Peric-Hupkes D, Meuleman W, Pagie L, Bruggeman SWM, Solovei I, Brugman W, et al (2010) Molecular Maps of the Reorganization of Genome-Nuclear Lamina Interactions during Differentiation. Molecular Cell 38: 603–613. 10.1016/j.molcel.2010.03.016 20513434 PMC5975946

[44] Lever E, Sheer D (2010) The role of nuclear organization in cancer. Journal of Pathology 220: 114–125. 10.1002/path.2651 19927301

[45] Bustamante C, Marko JF, Siggia ED, Smith S (1994) Entropic Elasticity of Lambda-Phage DNA. Science 265: 1599–1600. 8079175 10.1126/science.8079175

[46] Boer DR, Canals A, Coll M (2009) DNA-binding drugs caught in action: the latest 3D pictures of drug-DNA complexes. Dalton Transactions: 399–414. 10.1039/b809873p 19122895

[47] Zimmer C, Wahnert U (1986) Nonintercalating DNA-Binding Ligands—Specificity of the Interaction and Their Use as Tools in Biophysical, Biochemical and Biological Investigations of the Genetic Material. Progress in Biophysics & Molecular Biology 47: 31–112. 2422697 10.1016/0079-6107(86)90005-2

[48] Labarca C, Paigen K (1980) A simple, rapid, and sensitive DNA assay procedure. Analytical Biochemistry 102: 344–352. 6158890 10.1016/0003-2697(80)90165-7

[49] Tseng Y, Lee JS, Kole TP, Jiang I, Wirtz D (2004) Micro-organization and visco-elasticity of the interphase nucleus revealed by particle nanotracking. J Cell Sci 117: 2159–2167. 15090601 10.1242/jcs.01073

[50] Platani M, Goldberg I, Lamond AI, Swedlow JR (2002) Cajal body dynamics and association with chromatin are ATP-dependent. Mol Biol Cell 13: 376a–376a. 10.1038/ncb809 12068306

[51] Koche RP, Smith ZD, Adli M, Gu HC, Ku MC, Gnirke A, et al (2011) Reprogramming Factor Expression Initiates Widespread Targeted Chromatin Remodeling. Cell Stem Cell 8: 96–105. 10.1016/j.stem.2010.12.001 21211784 PMC3220622

[52] Zink D, Fischer AH, Nickerson JA (2004) Nuclear structure in cancer cells. Nature Reviews Cancer 4: 677–687. 15343274 10.1038/nrc1430

[53] Misteli T (2010) Higher-order Genome Organization in Human Disease. Cold Spring Harbor Perspectives in Biology 2. 10.1101/cshperspect.a000794 PMC2908770 20591991

[54] Yaron PN, Holt BD, Short PA, Losche M, Islam MF, Dahl KN (2011) Single wall carbon nanotubes enter cells by endocytosis and not membrane penetration. J Nanobiotechnology 9: 45 10.1186/1477-3155-9-45 21961562 PMC3195092

