# Correction: Spatially Resolved Quantification of Chromatin Condensation through Differential Local Rheology in Cell Nuclei Fluorescence Lifetime Imaging

**DOI:** 10.1371/journal.pone.0154639

**Published:** 2016-04-25

**Authors:** Stephen T. Spagnol, Kris Noel Dahl

Figs 1, 2, 3 and 4 are incorrect. There are discrepancies in the colors, and this has led to errors in the color key as well as missing data. The authors have provided a corrected version of each figure here.

**Fig 1:**
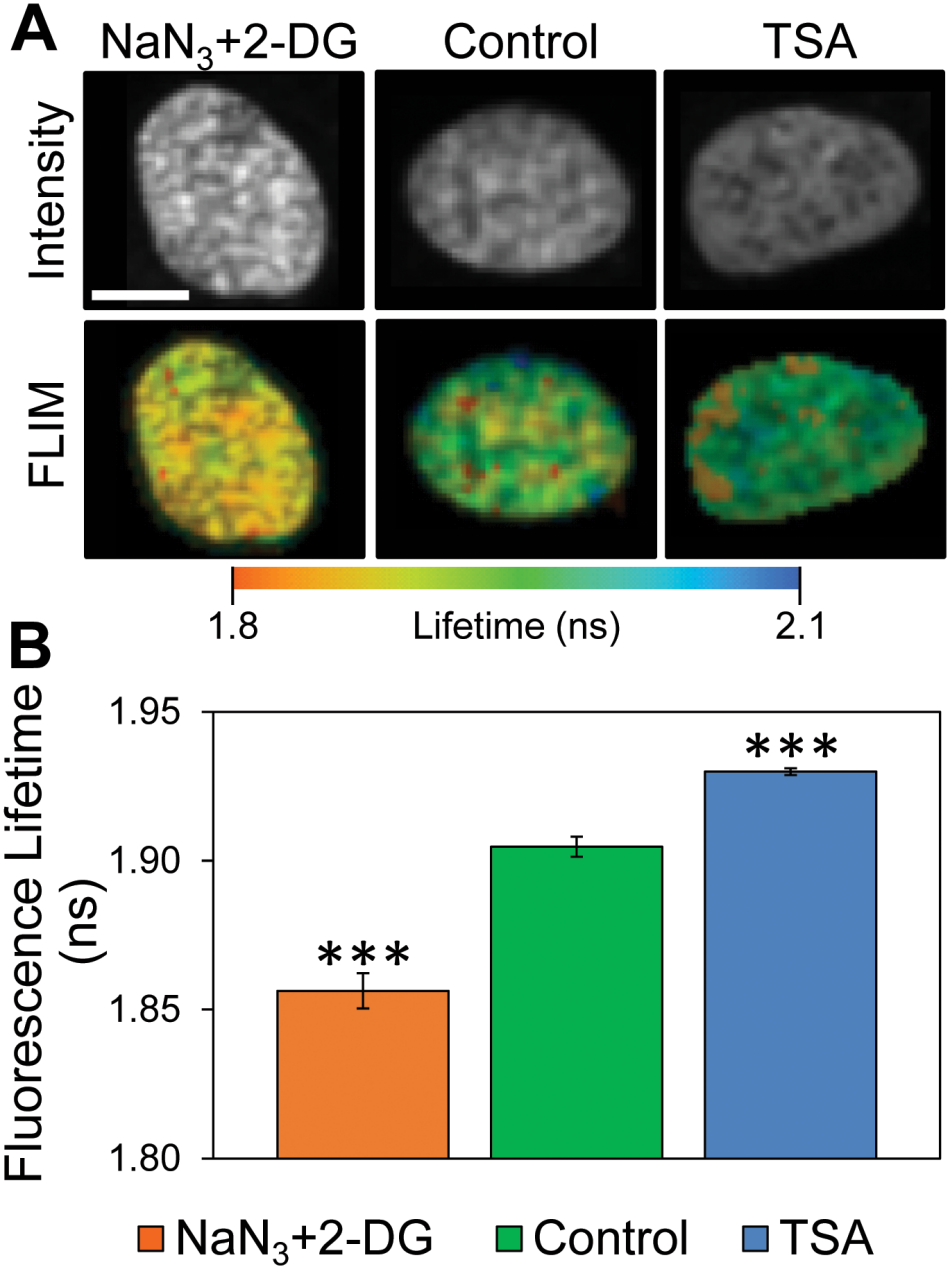
Fluorescence lifetime measurements of chromatin condensation state in human umbilical vein endothelial cell nuclei. (A) Fluorescence intensity confocal images (top) and mean fluorescence lifetime heat maps (bottom) of chromatin is measured in endothelial cell nuclei labeled with Hoechst 33342. Cells are treated with NaN_3_+2-DG for chromatin hypercondensation or with TSA for chromatin decondensation. Altered fluorescence intensity with treatments show differential chromatin condensation state, with more intense fluorescence arising from highly concentrated condensed chromatin. Mean fluorescence lifetime heat maps similarly indicate spatial arrangement of local fluorophore environments for labeled chromatin consistent with varying chromatin condensation state. Treatment with NaN_3_+2-DG results in more punctate regions of fluorescence intensity and shorter mean fluorescence lifetime (orange) relative to untreated controls, while TSA resulted in a significant reduction in punctate regions and longer mean fluorescence lifetime (blue). Scale bar is 10 μm. (B) The mean fluorescence lifetime of segmented nuclei for the various treatment conditions was calculated using Eq 2. Treatment with NaN_3_+2-DG resulted in a strong reduction in the mean fluorescence lifetime relative to untreated controls. By contrast, TSA treatment resulted in a dramatic increase in the mean fluorescence lifetime relative to untreated controls which indicated an increase in chromatin condensation state homogeneity throughout the cell nucleus. Error bars indicate standard error of the mean of pixel-to-pixel mean fluorescence lifetime differences of segmented nuclei in fields of view across multiple fields of view under each treatment condition (*** p<<0.001). Histograms and standard deviations are in S2 File.

**Fig 2:**
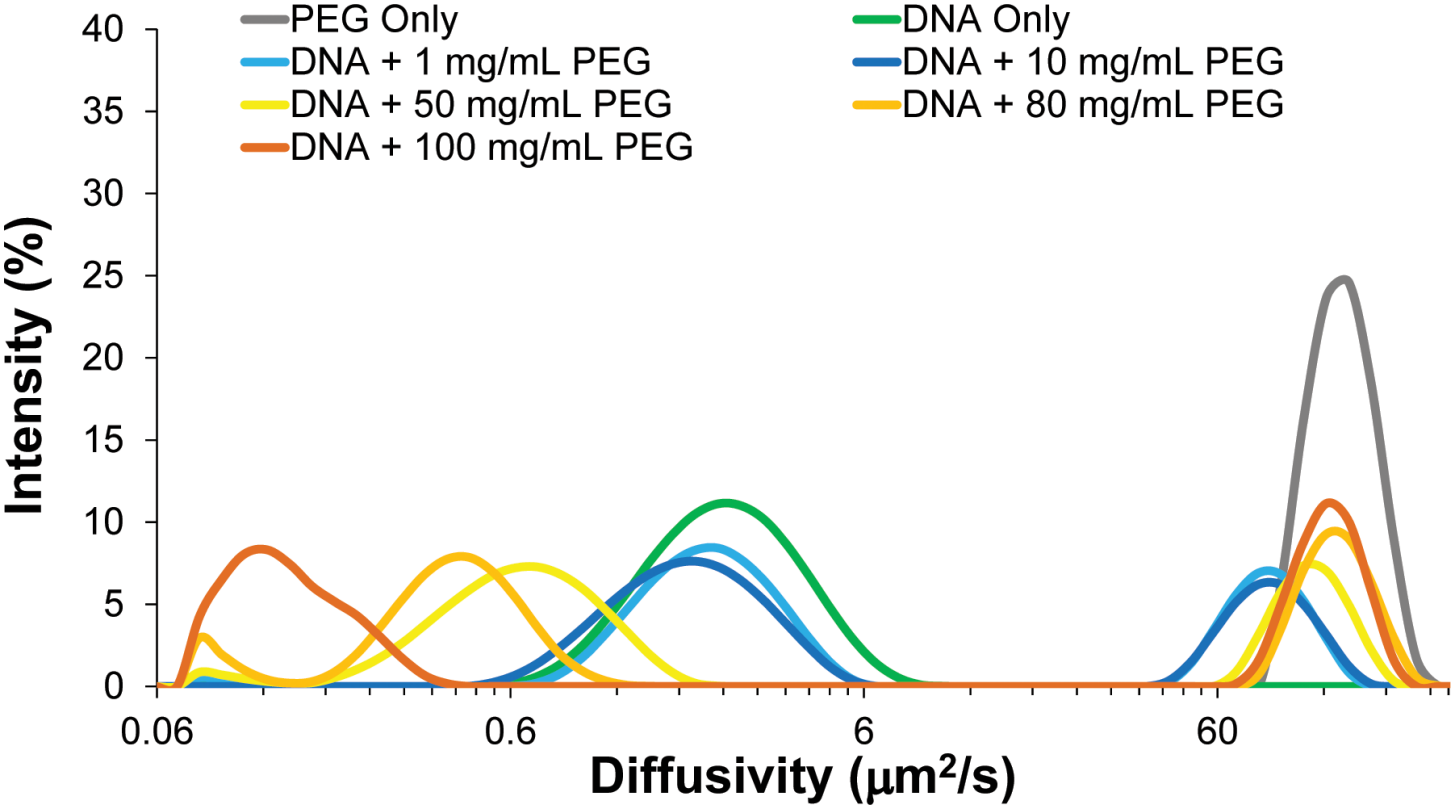
Dynamic light scattering measurements of in vitro λ-DNA solutions of varying condensation state. Measurements of PEG 6000 (gray) and λ-DNA (green) alone indicate their location within the combined solutions. As we increase PEG concentration, initially we see a negligible effect on the λ-DNA diffusivity distribution (shades of blue). At 50 mg/mL, the solution is above a threshold concentration of PEG 6000 and we observe a reduction in the λ-DNA diffusivity distribution, including a sharp decrease beyond the overlap concentration for PEG 6000 at 100 mg/mL (shades of yellow-orange). The initial reduction stems from the polymer-and-salt-induced (psi or ψ) condensation by macromolecular crowding-induced depletion forces. We show the regime over which λ-DNA is condensed and decondensed along with the location of the PEG population. Distributions are derived from 10–15 runs per individual measurements and averages of several measurements.

**Fig 3:**
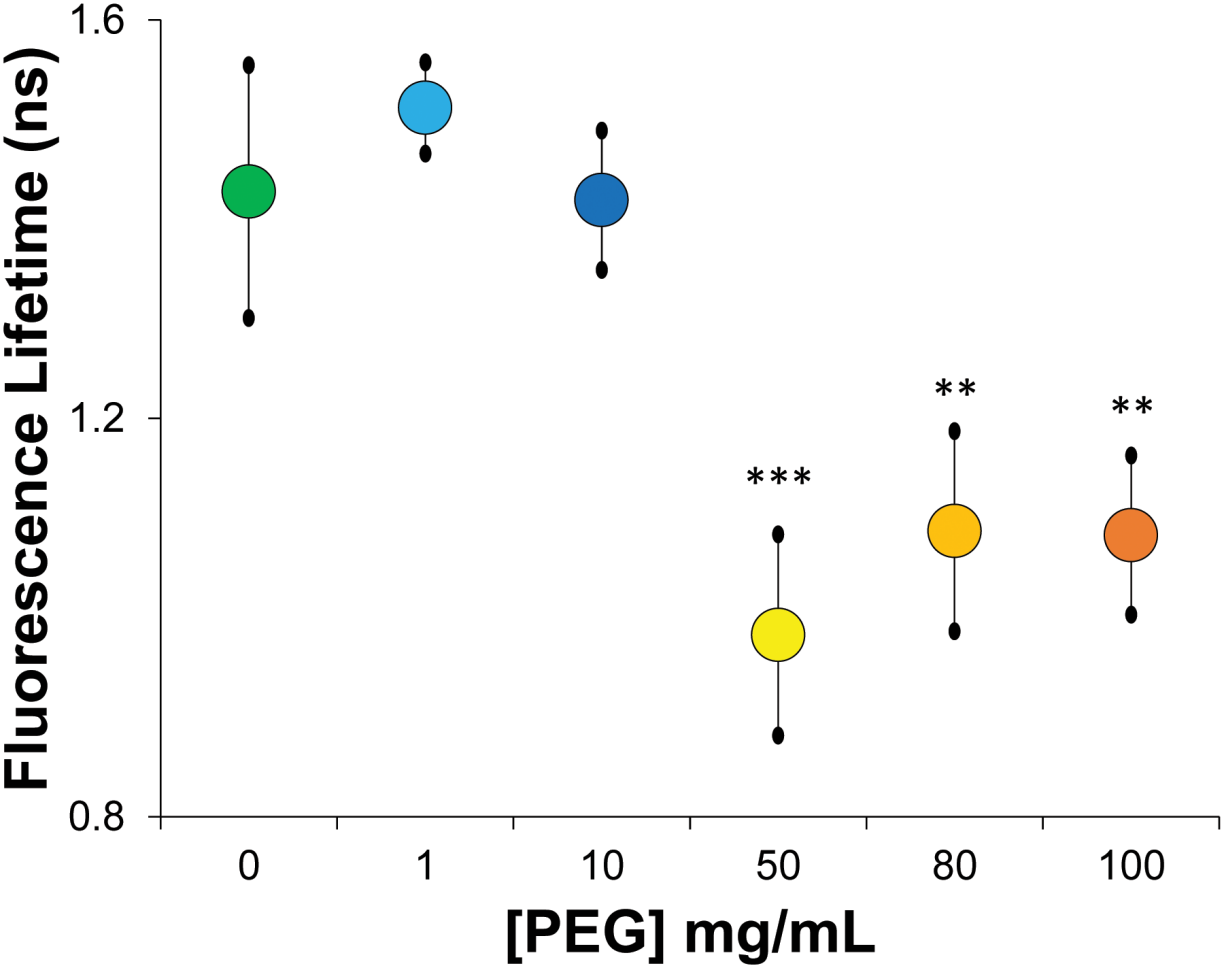
Fluorescence lifetime measurements of in vitro λ-DNA solutions of varying condensation state. As in the DLS experiments, we observe a dramatic reduction in the mean fluorescence lifetime above the threshold PEG 6000 concentration (~50 mg/mL; p<0.01) that is maintained at higher concentrations (shades of yellow-orange symbols). Interestingly, despite the increase in viscosity that occurs with increasing PEG concentration (including the sharp increase in trend above the overlap concentration at 100 mg/mL) we see no further statistical change in the mean fluorescence lifetime despite the dependence of the fluorescence lifetime on local viscosity. Error bars reflect standard deviation. Statistical significance based on Student’s t-test with the 0 mg/mL PEG, with **p<0.025 and ***p<0.01.

**Fig 4:**
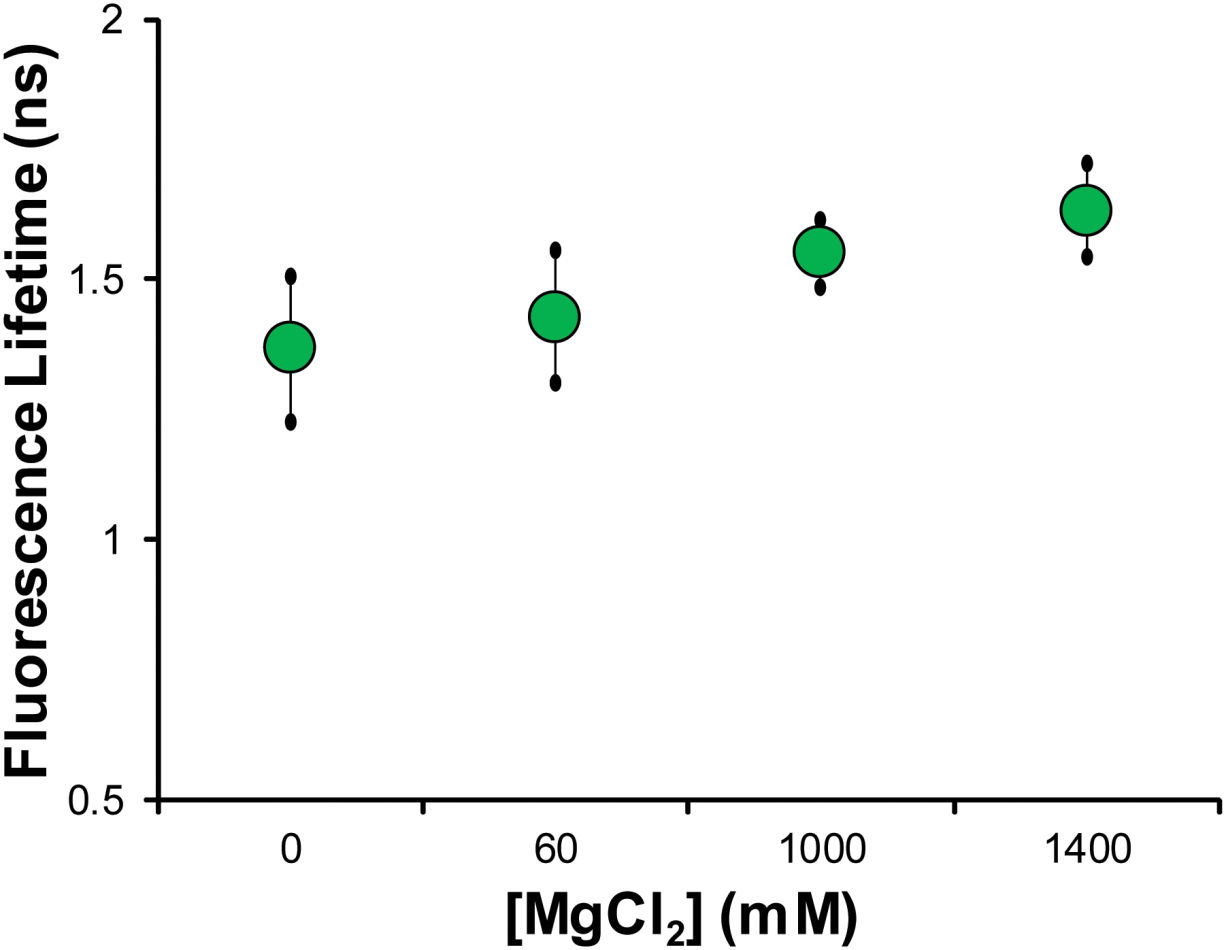
Fluorescence lifetime measurements of in vitro λ-DNA solutions of varying ionic strength solutions. The mean fluorescence lifetime of solutions of λ-DNA with varying concentration of MgCl_2_ shows no statistical dependence on ionic strength. Across a wide distribution of salt concentration varying over three orders of magnitude we see no statistically significant effect on the mean fluorescence lifetime, indicating it is not strongly influenced by salt concentration. Statistical comparisons made by Student’s t-test, with no statistical difference between solutions.

Specifically in Fig 2, the key marked as dark blue contains an error. The correct value should be as follows: “DNA + 10 mg/mL PEG”

## References

[1] Spagnol ST, Dahl KN (2016) Spatially Resolved Quantification of Chromatin Condensation through Differential Local Rheology in Cell Nuclei Fluorescence Lifetime Imaging. PLoS ONE 11(1): e0146244 doi: 10.1371/journal.pone.0146244 26765322 10.1371/journal.pone.0146244 PMC4713418

