# Supplementary material for: Spatially Resolved Quantification of Chromatin Condensation through Differential Local Rheology in Cell Nuclei Fluorescence Lifetime Imaging
Source: PLoS One. 2016 Jan 14;11(1):e0146244. doi: 10.1371/journal.pone.0146244 (PMC4713418; doi:10.1371/journal.pone.0146244)
Supplement: S1 File — Fig A. Fluorescence lifetime heat maps of chromatin is measured in endothelial cell nuclei labeled with Hoechst 33342. Fixed samples, similar to those used throughout a majority of the results, show a spatial distribution of lifetimes. Live cells also show a spatial distribution of lifetimes in the same regime. Fig B. The mean fluorescence lifetime of segmented nuclei for the various treatment conditions was calculated using Eq 2. Samples were statistically similar with one another. Live cells showed greater variation likely from exchange of dye between the nucleus and media as well as cell motility. (DOCX) [file pone.0146244.s001.docx]

**
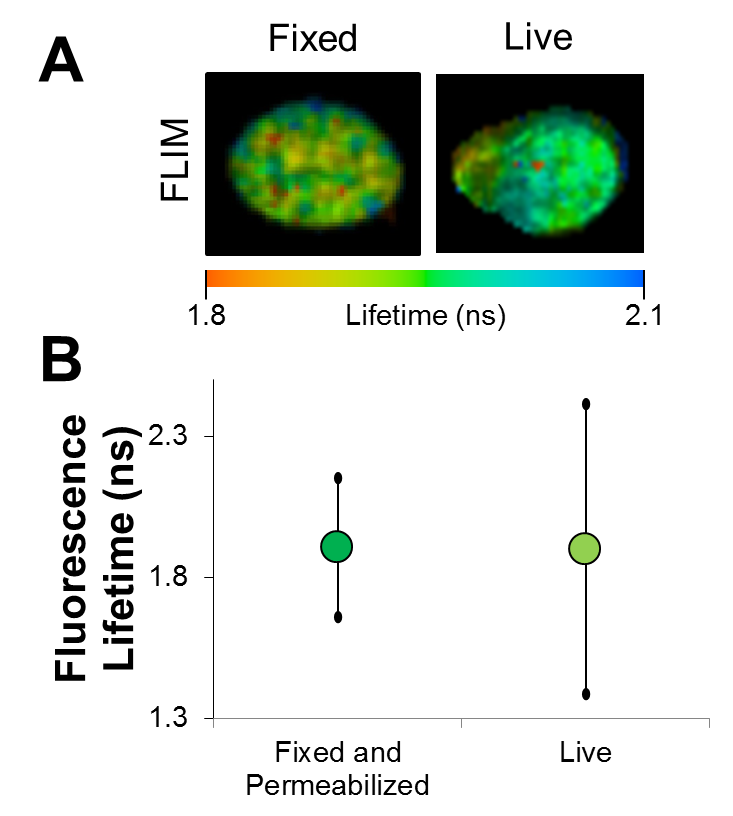
**

**S1 File: Fluorescence lifetime measurements of chromatin condensation state in fixed and live human umbilical vein endothelial cell nuclei.**

**Fig A.** Fluorescence lifetime heat maps of chromatin is measured in endothelial cell nuclei labeled with Hoechst 33342. Fixed samples, similar to those used throughout a majority of the results, show a spatial distribution of lifetimes. Live cells also show a spatial distribution of lifetimes in the same regime.

**Fig B.** The mean fluorescence lifetime of segmented nuclei for the various treatment conditions was calculated using Equation 2. Samples were statistically similar with one another. Error bars indicate standard deviation of pixel-to-pixel mean fluorescence lifetime differences of segmented nuclei in fields of view across multiple fields of view under each treatment condition. Live cells showed greater variation likely from exchange of dye between the nucleus and media as well as cell motility.
