# Supplementary material for: Spatially Resolved Quantification of Chromatin Condensation through Differential Local Rheology in Cell Nuclei Fluorescence Lifetime Imaging
Source: PLoS One. 2016 Jan 14;11(1):e0146244. doi: 10.1371/journal.pone.0146244 (PMC4713418; doi:10.1371/journal.pone.0146244)
Supplement: S2 File — Fig A. Histograms of lifetimes are shown for each condition to highlight the spatial heterogeneity for each sample. NaN3+2-DG treatment shows a significant contribution from very low lifetimes (0.6 ns and 1.3). This reduces the mean fluorescence lifetime (dashed line). In contrast, the mean fluorescence lifetime of TSA treated nuclei is increased with a smaller variance since there is little contribution from very low lifetimes. Fig B. The mean fluorescence lifetime of segmented nuclei for the various treatment conditions was calculated using Eq 2. Treatment with NaN3+2-DG resulted in a strong reduction in the mean fluorescence lifetime relative to untreated controls. By contrast, TSA treatment resulted in a dramatic increase in the mean fluorescence lifetime relative to untreated controls as well as a large reduction in the variance which indicated an increase in chromatin condensation state homogeneity throughout the cell nucleus. Error bars indicate standard deviation of pixel-to-pixel mean fluorescence lifetime differences of segmented nuclei in fields of view across multiple fields of view under each treatment condition. (DOCX) [file pone.0146244.s002.docx]

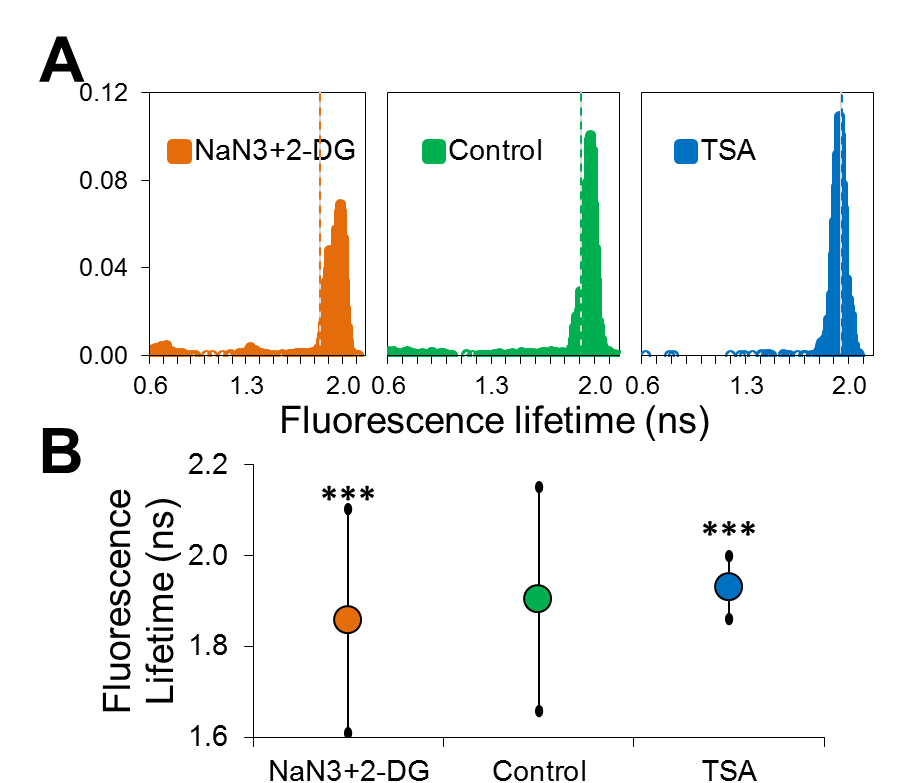


**S2 File: Distributions of fluorescence lifetime in nuclei of cells with different treatments.**

**Fig A.** Histograms of lifetimes are shown for each condition to highlight the spatial heterogeneity for each sample. NaN_3_+2-DG treatment shows a significant contribution from very low lifetimes (0.6 ns and 1.3). This reduces the mean fluorescence lifetime (dashed line). In contrast, the mean fluorescence lifetime of TSA treated nuclei is increased with a smaller variance since there is little contribution from very low lifetimes. Analysis was done using 60-80 segmented nuclei for each treatment condition.

**Fig B.** The mean fluorescence lifetime of segmented nuclei for the various treatment conditions was calculated using Equation 2. Treatment with NaN_3_+2-DG resulted in a strong reduction in the mean fluorescence lifetime relative to untreated controls (p<<0.001). By contrast, TSA treatment resulted in a dramatic increase in the mean fluorescence lifetime relative to untreated controls (p<<0.001) as well as a large reduction in the variance (p<<0.001) which indicated an increase in chromatin condensation state homogeneity throughout the cell nucleus. Error bars indicate standard deviation of pixel-to-pixel mean fluorescence lifetime differences of segmented nuclei in fields of view across multiple fields of view under each treatment condition. Standard deviation was used in place of standard error of the mean to emphasize the reduction in the fluorescence lifetime variance from chromatin decondensation from TSA treatment (p<<0.001).
