# Supplementary material for: Spatially Resolved Quantification of Chromatin Condensation through Differential Local Rheology in Cell Nuclei Fluorescence Lifetime Imaging
Source: PLoS One. 2016 Jan 14;11(1):e0146244. doi: 10.1371/journal.pone.0146244 (PMC4713418; doi:10.1371/journal.pone.0146244)
Supplement: S3 File — Fig A. Fluorescence intensity confocal images and mean fluorescence lifetime heat maps of chromatin is measured in endothelial cell nuclei labeled with PicoGreen. Cells are treated with TSA for chromatin decondensation. Altered fluorescence intensity with treatments show differential chromatin condensation state, with more intense fluorescence arising from highly concentrated condensed chromatin. Mean fluorescence lifetime heat maps similarly indicate spatial arrangement of local fluorophore environments for labeled chromatin consistent with varying chromatin condensation state. Treatment with TSA resulted in a significant reduction in punctate regions and longer mean fluorescence lifetime relative to untreated controls. Fig B. The mean fluorescence lifetime of segmented nuclei for the various treatment conditions was calculated using Eq 2. Treatment with TSA treatment resulted in a dramatic increase in the mean fluorescence lifetime relative to untreated controls as well as a large reduction in the variance which indicated an increase in chromatin condensation state homogeneity throughout the cell nucleus. Error bars indicate standard deviation of pixel-to-pixel mean fluorescence lifetime differences of segmented nuclei in fields of view across multiple fields of view under each treatment condition. Standard deviation was used in place of standard error of the mean to emphasize the reduction in the fluorescence lifetime variance from chromatin decondensation from TSA treatment. (DOCX) [file pone.0146244.s003.docx]

**
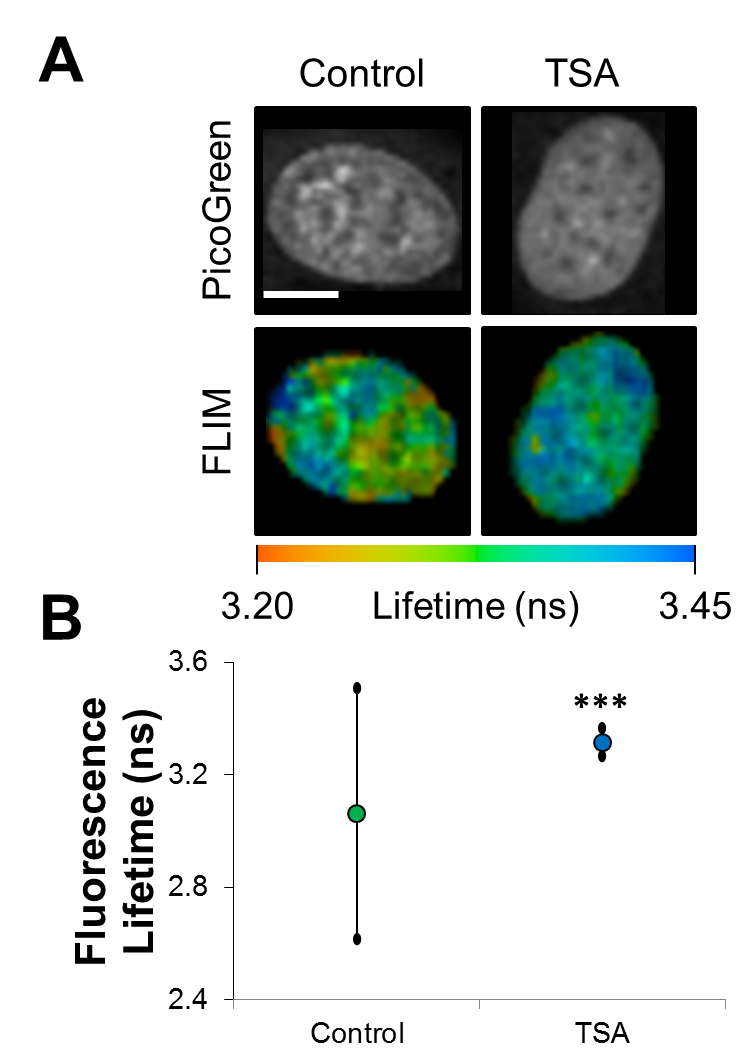
**

**S3 File: Fluorescence lifetime measurements of chromatin condensation state in human umbilical vein endothelial cell nuclei with PicoGreen.**

**Fig A.** Fluorescence intensity confocal images (top) and mean fluorescence lifetime heat maps (bottom) of chromatin is measured in endothelial cell nuclei labeled with PicoGreen. Cells are treated with TSA for chromatin decondensation. Altered fluorescence intensity with treatments show differential chromatin condensation state, with more intense fluorescence arising from highly concentrated condensed chromatin. Mean fluorescence lifetime heat maps similarly indicate spatial arrangement of local fluorophore environments for labeled chromatin consistent with varying chromatin condensation state. Treatment with TSA resulted in a significant reduction in punctate regions and longer mean fluorescence lifetime (blue) relative to untreated controls. Scale bar is 10 μm.

**Fig B.** The mean fluorescence lifetime of segmented nuclei for the various treatment conditions was calculated using Equation 2. Treatment with TSA treatment resulted in a dramatic increase in the mean fluorescence lifetime relative to untreated controls (p<<0.001) as well as a large reduction in the variance (p<<0.001) which indicated an increase in chromatin condensation state homogeneity throughout the cell nucleus. Error bars indicate standard deviation of pixel-to-pixel mean fluorescence lifetime differences of segmented nuclei in fields of view across multiple fields of view under each treatment condition. Standard deviation was used in place of standard error of the mean to emphasize the reduction in the fluorescence lifetime variance from chromatin decondensation from TSA treatment (p<<0.001).
